# Deciphering preferential interactions within supramolecular protein complexes: the proteasome case

**DOI:** 10.15252/msb.20145497

**Published:** 2015-01-05

**Authors:** Bertrand Fabre, Thomas Lambour, Luc Garrigues, François Amalric, Nathalie Vigneron, Thomas Menneteau, Alexandre Stella, Bernard Monsarrat, Benoît Van den Eynde, Odile Burlet-Schiltz, Marie-Pierre Bousquet-Dubouch

**Affiliations:** 1 CNRS, IPBS (Institut de Pharmacologie et de Biologie Structurale)Toulouse, France; 2 Université de Toulouse, UPS, IPBSToulouse, France; 3 Ludwig Institute for Cancer ResearchBrussels, Belgium; 4 WELBIO (Walloon Excellence in Life Sciences and Biotechnology)Brussels, Belgium; 5 de Duve Institute, Université catholique de LouvainBrussels, Belgium

**Keywords:** affinity purification, correlation profiling, label-free quantitative proteomics, mass spectrometry

## Abstract

In eukaryotic cells, intracellular protein breakdown is mainly performed by the ubiquitin–proteasome system. Proteasomes are supramolecular protein complexes formed by the association of multiple sub-complexes and interacting proteins. Therefore, they exhibit a very high heterogeneity whose function is still not well understood. Here, using a newly developed method based on the combination of affinity purification and protein correlation profiling associated with high-resolution mass spectrometry, we comprehensively characterized proteasome heterogeneity and identified previously unknown preferential associations within proteasome sub-complexes. In particular, we showed for the first time that the two main proteasome subtypes, standard proteasome and immunoproteasome, interact with a different subset of important regulators. This trend was observed in very diverse human cell types and was confirmed by changing the relative proportions of both 20S proteasome forms using interferon-γ. The new method developed here constitutes an innovative and powerful strategy that could be broadly applied for unraveling the dynamic and heterogeneous nature of other biologically relevant supramolecular protein complexes.

## Introduction

The proteasome is a supramolecular protein machinery that is central to protein homeostasis. In all eukaryotic cells, it is involved in the selective degradation of most short-lived intracellular proteins (Hershko & Ciechanover, 1998; Glickman & Ciechanover, 2002), ensuring subtle modulation of gene expression, but also the removal of misfolded, aberrant (i.e. oxidized or mutated), or otherwise damaged proteins, avoiding cytotoxicity. In higher eukaryotes, it is critical to the immune response because it generates antigenic peptide precursors that can be recognized by cytotoxic T lymphocytes (CTLs) on MHC class I molecules.

Proteasome particles are formed by the dynamic association of several sub-complexes, a 20S core particle (20S CP), either single or associated with one or two regulatory particles (RPs) of identical or different protein composition. Proteasome complexes thus display a high degree of heterogeneity. The 20S CP presents a α7β7β7α7 barrel-like structure and was shown to exist in the eukaryotic cell as four different subtypes, depending on the subsets of incorporated catalytic beta subunits. In the standard proteasome (sP20S), the two β rings each contain three standard catalytic subunits, β1, β2, and β5, which are replaced by distinct immunosubunits, β1i, β2i, and β5i, in the immunoproteasome (iP20S), respectively. Two intermediate 20S CP subtypes, β5i 20S proteasome (β5i P20S) and β1iβ5i 20S proteasome (β1iβ5i P20S), bearing a mixed incorporation of standard and immunosubunits, β1, β2, β5i and β1i, β2, β5i, respectively, have also been identified in a wide range of cell types and tissues (Guillaume *et al*, 2010). The catalytic subunits are responsible for the three proteasome proteolytic activities (trypsin like, chymotrypsin like, and caspase like), which can be modulated by the replacement of standard subunits by immunosubunits (Orlowski & Wilk, 2000; Basler *et al*, 2013). The iP20S is induced during the immune response in mammals but also exists in various amounts as constitutive proteasome complexes, depending on tissues or cell type (Dahlmann *et al*, 2000; Zoeger *et al*, 2006; Klare *et al*, 2007; Bousquet-Dubouch *et al*, 2008; Guillaume *et al*, 2010, 2012). The two α rings are located at the opposite ends of the proteolytic cavity and regulate the access of substrates to catalytic sites through gated pores of 13 Å in diameter. In mammals, gate opening can be efficiently triggered through the association of the 20S CP with four main different RPs, the 19S regulatory particle (19S RP), PA28αβ, PA28γ (otherwise known as 11S RPs), and PA200. One 20S CP can interact at its two sides with either two identical regulators or two different ones, thus forming hybrid proteasomes (Tanahashi *et al*, 2000). The most studied regulator, the 19S RP, is involved in the recognition, the unfolding, and the translocation of poly-ubiquitinated substrates into the 20S CP for degradation. In addition to the 19S RP, PA28αβ and PA28γ RPs are abundant 20S proteasome-associated regulators present in the cytosol and the nucleus, respectively (Drews *et al*, 2007; Fabre *et al*, 2013). They catalyze protein degradation through an ubiquitin-independent pathway, which still needs to be completely clarified (Stadtmueller & Hill, 2011; Kish-Trier & Hill, 2013).

Although the binding constants between the different 20S subtypes and its different RPs are not known, the binding mode between the α-ring and RPs has been established precisely by numerous structural studies and was found to be shared among species (Stadtmueller & Hill, 2011; Beck *et al*, 2012; da Fonseca *et al*, 2012; Lander *et al*, 2012; Lasker *et al*, 2012; Kish-Trier & Hill, 2013). In all cases, it involves the C-termini of RP subunits and a pocket at the interface between α-subunits. Recently, in-solution NMR surveys have evidenced an allosteric pathway linking the binding sites of C-termini of the 11S RP with the active sites of the *Thermoplasma acidophilum* 20S CP, emphasizing a clear connection between these regions that are 80 Å apart. In particular, the modification of active sites in the *T. acidophilum* CP was shown to induce structural changes at the α-ring binding interface (Ruschak & Kay, 2012). This clearly suggests that changes in active sites configurations, as found in sP20S and iP20S, might affect binding affinities for RPs. It would thus be of great interest to characterize proteasome heterogeneity and to determine whether preferential associations within proteasome sub-complexes do exist.

Affinity purification coupled to mass spectrometry (AP-MS) is a very powerful and sensitive approach for the determination of protein complexes composition through the specific capture of a target protein and all associated partners (Gingras *et al*, 2007; Trinkle-Mulcahy *et al*, 2008; Glatter *et al*, 2009). A few studies take advantage of the quantitative nature of affinity purification associated with mass spectrometry (AP-MS) data to define network architecture (Choi *et al*, 2010; Lee *et al*, 2011). However, in most cases, interactome approaches using multiple baits are performed in a given biological context. Moreover, they cannot easily resolve the distribution of a protein of interest among the different complexes in which it might be embedded (Zaki & Mora, 2014). System-wide studies of the composition and dynamics of protein complexes have recently been addressed using an alternative method to AP-MS, protein correlation profiling associated with mass spectrometry (PCP-MS). This approach involves biochemical fractionation procedures and allows the assignment of proteins to specific organelles (Andersen *et al*, 2003; Foster *et al*, 2006; Gatto *et al*, 2010) or, more recently, to protein complexes (Kristensen *et al*, 2012), by comparing the proteins elution profiles acquired by quantitative MS. As far as interactome studies are concerned, PCP-MS increases the analysis throughput of protein complexes dynamics (Kristensen *et al*, 2012) and also helps monitoring the impact of subunit isoforms or post-translational modifications in multiprotein complexes (Kirkwood *et al*, 2013). Interestingly, the heterogeneity of proteasomes can, at least in part, be resolved using PCP-MS on size exclusion chromatography (SEC)-separated protein complexes because this approach is able to distinguish and quantify the relative proportions of singly and doubly capped 20S CPs (Kristensen *et al*, 2012).

The aim of the present study was to decipher proteasome heterogeneity through modern label-free quantitative proteomics. Using PCP-MS on glycerol gradient-separated proteasome complexes, we could first reveal a previously unreported preferential association of immunoproteasome (iP20S) with the PA28αβ RP. Then through the development of a new workflow combining PCP-MS and AP-MS, we could increase the sensitivity of detection of proteasome regulators and thus go deeper into proteasome characterization. Indeed, by correlating proteins abundances across a large set of 24 proteasome samples immunopurified from nine different human cell lines, we observed that the two main 20S proteasome subtypes, sP20S and iP20S, interact with a different subset of regulators. Some of these preferential interactions were validated by artificially or physiologically changing the proportions of both 20S CP subtypes in assembled proteasomes. This novel integrated proteomic workflow provides a valuable tool to better understand the dynamic and complex nature of molecular systems.

## Results

### PCP-MS analysis of glycerol density gradient-separated proteasome complexes

**Figure 1:**
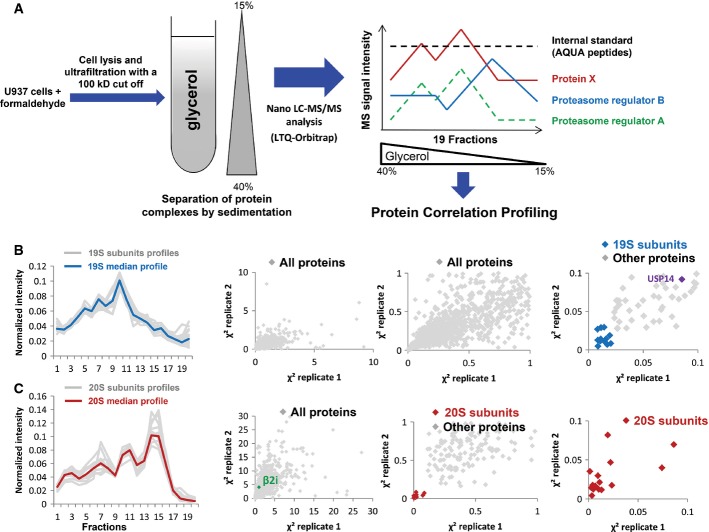
Protein correlation profiling (PCP) analysis of glycerol density gradient-separated proteasome complexes

**Figure 2:**
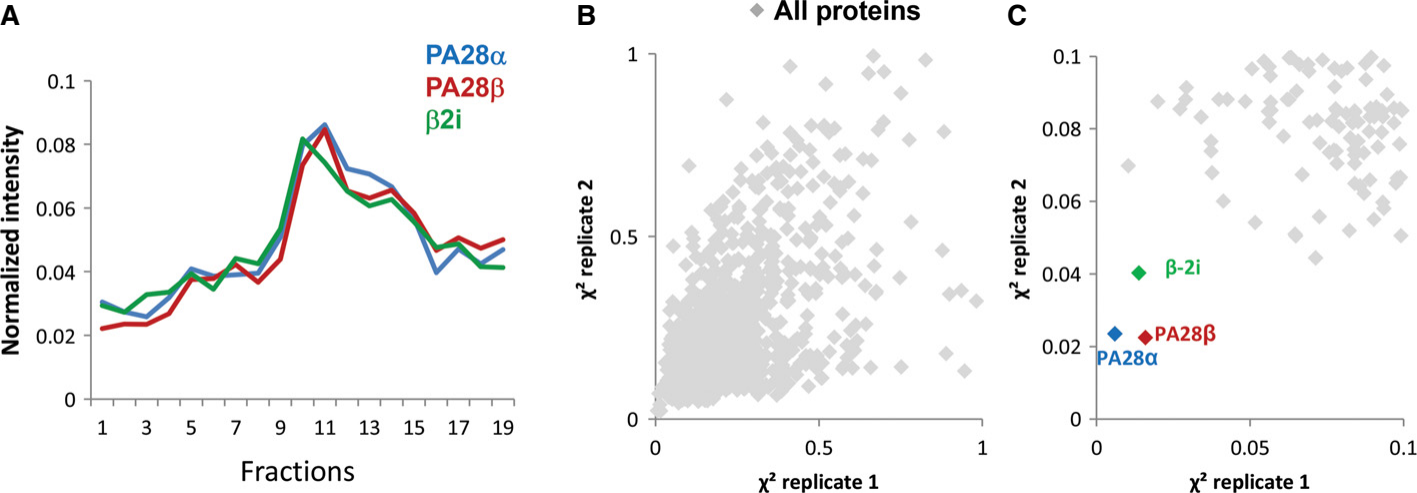
Protein correlation profiling (PCP) analysis using the median profile of the PA28αβ regulator as the reference profile

In a first attempt to resolve proteasome complexes heterogeneity and identify components of the different proteasome subtypes, we performed a PCP-MS analysis on U937 AML cell proteins separated by glycerol density gradient ultracentrifugation (Fig1A). This cell line is particularly well suited for the analysis of proteasome diversity because it contains equal amounts of each 20S proteasome subtype and, in particular, very similar quantities of β5 and β2i catalytic subunits (Fabre *et al*, 2013), which are uniquely found in sP20S and iP20S complexes, respectively (Guillaume *et al*, 2010). To maintain proteasome integrity throughout the purification process, cells were cross-linked *in vivo* with formaldehyde. After cell lysis, low-MW proteins (below 100 kDa) were discarded by ultrafiltration so that a high-quality separation of high-MW protein complexes could be performed on a glycerol density gradient. Proteins participating in the different complexes resolved in each fraction of the density gradient were then identified and quantified using high-resolution mass spectrometry analysis coupled online to liquid chromatography. Label-free quantification based on peptide ion extracted chromatograms was performed (Mouton-Barbosa *et al*, 2010; Gautier *et al*, 2012) using the TOP3 quantification method (Silva *et al*, 2006). A protein abundance index (PAI) was calculated to approximate the relative quantity of each proteasome subunit and proteasome-associated protein. To handle the inter-run signal variations, heavy internal standards, composed of eight peptides containing isotopically labeled arginine or lysine and eluting all along the chromatographic gradient, were added into each sample before injection. A MS-based intensity profile could therefore be obtained for the 3,353 proteins identified and quantified in the 19 fractions of two biological replicates (Supplementary Table S1). The profiles obtained for the 16 identified subunits of the 19S regulator (Rpt1–6, Rpn1–3, Rpn5, 7–9, 11–13) (Fig1B, left panel) and for the 11 different non-catalytic subunits of the 20S proteasome (α1–α7, β3, β4, β6, and β7) (Fig1C, left panel) showed a low dispersion from their respective median profiles, validating the method. Interestingly, the two median profiles of all subunits corresponding to the 20S CP and the 19S RP were somewhat different, in particular in low density fractions 13–16. The high signal detected for the 20S subunits in this profile area corresponds to free 20S core particle elution fractions (no 19S subunits detected) and confirms our previous results showing that a large proportion of 20S proteasome is present as a free particle in the U937 cell line (Fabre *et al*, 2013). To screen for possible protein interaction among the same complexes, we calculated a relative Euclidian distance, defined in the experimental section and called χ^2^, between a reference profile and the profile of all the proteins identified in the gradient. This statistical method has proven its efficiency for comparison of sedimentation protein profiles in quantitative proteomic experiments (Andersen *et al*, 2003; Wiese *et al*, 2007). The distances obtained from two independent gradient experiments performed on two biological replicates were then plotted to further increase the confidence in protein complexes assignments. When the 19S median profile was taken as reference, all subunits from this protein complex exhibited low χ^2^ values, under 0.05, and were accordingly gathered in an area very close to the origin of the graph (Fig1B, middle and right panels). Usp14, a known 19S interacting deubiquitinating enzyme, was also observed at a very close distance (mean χ^2^ = 0.09), as expected (Fig1B, right panel). We then applied the PCP-MS analysis to 20S proteasome subunits and observed that the 20S subunits distribution could be clearly distinguished from the other proteins identified in the gradient fractions (Fig1C, 3^rd^ panel). Unexpectedly, the 20S immunocatalytic β2i subunit was the only 20S subunit observed at a very high distance from the other 20S subunits (mean χ^2^ = 3.5) (Fig1C, 2^nd^ panel). However, a close correlation was evidenced with both the PA28α and PA28β subunits of the PA28αβ complex, when the latter was taken as reference profile (Fig2A). The β2i immunocatalytic subunit is by far the protein showing the closest profile to that of PA28αβ, when compared to all the proteins quantified in the U937 glycerol sedimented lysate (χ^2^ mean value of 0.025) (Fig2B and C). As β2i is exclusively found in the iP20S (Guillaume *et al*, 2010), these data therefore suggest a so far unknown preferential association between the PA28αβ regulator and the iP20S.

PCP-MS strategy to identify proteins interacting with specific proteasome subtypes. U937 cells were cross-linked with formaldehyde and lysed, and proteins were concentrated and ultrafiltrated on a 100 kDa cutoff device. Protein complexes were then separated on a 15–40% glycerol gradient. Each fraction of the gradient was analyzed by nano-LC-MS/MS. Protein quantification was performed using the mean XIC of the three most intense validated peptides for each protein, after internal standard calibration using a mix of 8 isotopically labeled peptides. The PCP analysis was performed as described in the Materials and Methods section.

PCP analysis of the 19S regulatory complex. Protein abundance profiles of 16 proteins of the 19S RP (Rpt1–6, Rpn1–3, Rpn5, 7–9, 11–13, gray lanes) and of their median abundance (black lane) (left panel). PCP analysis is performed by plotting the χ^2^ values (representing the Euclidian distance between the abundance profile of each protein and the reference profile) of the experimental replicate 2 as a function of the χ^2^ values of the experimental replicate 1 (middle left panel). The median profile of the 19S complex subunits was used as the reference profile for the calculation of the χ^2^ values. Different zooms of the graph are represented (middle right and right panels). Light gray dots represent the proteins quantified in all the fractions of the density gradient and blue dots represent 19S subunits (right panel).

PCP analysis of proteasome 20S complex. Protein abundance profiles of 17 proteins of the 20S CP (α1–α7, β1–β7, β1i, β2i, β5i, gray lanes) and of their median abundance (black lane) (left panel). PCP analysis is performed by plotting the χ^2^ values of the experimental replicate 2 as a function of the χ^2^ values of the experimental replicate 1 (middle left panel). The median profile of the 20S complex subunits was used as the reference profile for the calculation of the χ^2^ values. Different zooms of the graph are represented (middle right and right panels). Light gray dots represent the proteins quantified in all the fractions of the density gradient and red dots represent 20S subunits.

Profiles of the PA28α, PA28β, and the β2i proteins (blue, red and green lines, respectively).

Plot of the χ^2^ values of the experimental replicate 2 as a function of the χ^2^ values of the experimental replicate 1.

A zoom of the graph in (B) is represented and χ^2^ coordinates for PA28α, PA28β, and β2i proteins are highlighted as blue, red, and green dots, respectively. Light gray dots represent the χ^2^ coordinates of the proteins quantified in all the fractions of the gradient. The median profile of the PA28α and PA28β subunits was used as the reference profile for the calculation of the χ^2^ values.

### The abundances of core subunits of proteasome sub-complexes strongly correlate in nine different human cell lines

**Figure 3:**
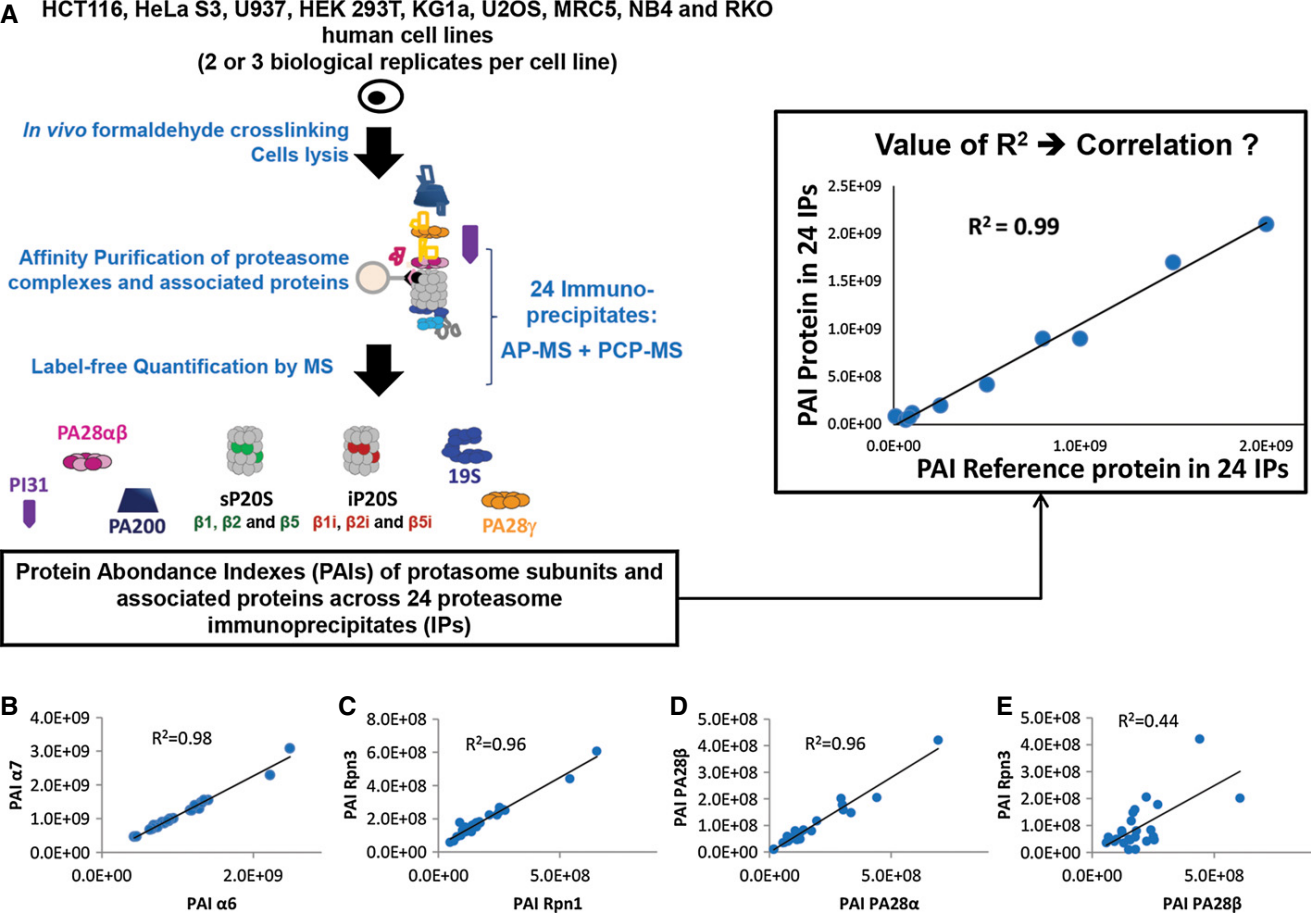
Protein abundance correlation of affinity-purified complexes analyzed by mass spectrometry strategy applied to proteasome complexes
A Proteasome complexes were immunopurified from nine formaldehyde-crosslinked human cell lines and analyzed by nano-LC-MS/MS. Protein abundance indexes (PAIs) were used to represent the abundance of proteins in purified proteasome samples. The correlation between two different proteins was quantified using coefficients of determination (*R*^2^).
B–E Correlations of abundances of α7 and α6 (B), Rpn3 and Rpn1 (C), PA28β and PA28α (D), and Rpn3 and PA28β (E).

To confirm the preferential association between the PA28αβ regulator and the iP20S and to allow a deeper characterization of proteasome complexes, proteasomes were affinity-purified from nine different formaldehyde cross-linked cell lines and analyzed by nano-LC-MS/MS, as previously described (Fabre *et al*, 2013). Formaldehyde cross-linking was shown to be required to stabilize the association of all regulatory particles with the 20S core particle (Fabre *et al*, 2013). To reach the largest diversity of proteasome complexes, we analyzed the proteolytic complex in a wide variety of human cell lines, including hematopoietic and epithelial cell lines of different origins and exhibiting high variations both in the composition of catalytic subunits and in the stoichiometry of bound regulators or other associated proteins. Very high 20S proteasome purification yields (87 ± 5%) could be obtained (Supplementary Fig S1A). A protein abundance index (PAI) was calculated for each proteasome subunit or proteasome-associated protein identified in the immunopurified complexes obtained from two or three biological replicates of each cell line (Fig3A and Materials and Methods section for details). Importantly, no averaging of biological replicates was performed, to keep the experimental variability. The abundances thus obtained for each protein were then compared pairwise with the ones of another protein, called reference protein, across the 24 proteasome immunoprecipitates (Fig3A). The correlation between the abundances of two pairwise proteins was estimated using the coefficient of determination (*R*^2^), which is more stringent than the usually used Pearson's correlation coefficient (*R*) (Kirkwood *et al*, 2013). Interestingly, when using the PAI as a relative abundance metric, very strong correlations were obtained between subunits belonging to the same complex, such as the 20S proteasome non-catalytic subunits α6 and α7 (*R*^2^ = 0.98), the 19S regulator subunits Rpn1 and Rpn3 (*R*^2^ = 0.96) or the PA28α and PA28β subunits (*R*^2^ = 0.96) that compose the PA28αβ activator (Fig3B–D). All 20S proteasome non-catalytic subunits (α1–α7, β3, β4, β6, and β7) (gathered in a group of proteins called ‘ncP20S’) or subunits belonging to the 19S regulator (Rpt1–6, Rpn1–3, 5–14) were pairwise compared and very high coefficients of determination (0.90 ± 0.07 and 0.93 ± 0.04, respectively) were obtained (Supplementary Fig S1B), demonstrating the efficiency of the AP-MS strategy used to correlate proteins belonging to the same complex. Moreover, the cellular expression levels of many 19S subunits and of some 20S subunits are not correlated (Supplementary Fig S1D), contrary to the abundances of these proteins in immunopurified proteasomes (Supplementary Fig S1B), showing that the immuno-enrichment step is required to highlight subunits interactions within a protein complex.

Conversely, when plotting the PAIs of Rpn3 and PA28β, proteins belonging to the 19S RP and the PA28αβ regulator, respectively, a much weaker correlation (*R*^2^ = 0.44) could be observed (Supplementary Fig S3). This is probably because the 19S RP is involved in several different types of functional proteasome complexes (30S, 26S for instance) in addition to hybrid proteasome (one 19S RP and one PA28 RP associated with one 20S CP), which could indeed be observed in U937 cells when immunopurifying PA28β (Supplementary Fig S1C).

These results therefore show that the protein abundance index, associated with the *R*^2^, is able to quantitatively describe the correlation of the relative abundances of proteins constituting core subunits of proteasome sub-complexes purified from a large set of human cell lines exhibiting a high variety of proteasome complexes. Comparing the abundances of the different proteasome subunits and associated proteins in purified proteasome preparations therefore appears as an efficient approach to unravel putative binary protein interactions among proteasome complexes.

### Proteasome subunits and associated proteins cluster differently on the basis of their abundances across the nine cell lines

**Figure 4:**
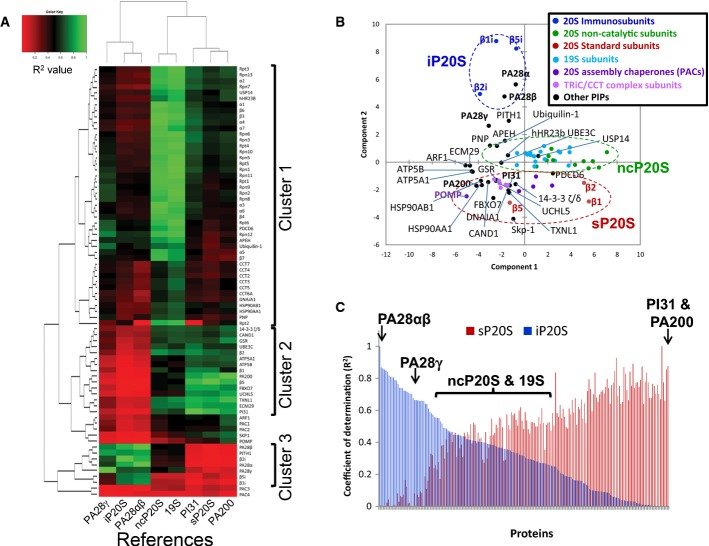
Protein abundance correlation of affinity-purified complexes analyzed by mass spectrometry analysis applied to the proteasome complexes and their interacting proteins

To investigate further the composition of proteasome complexes and highlight putative unknown interactions among specific proteasome subunits or associated proteins, we pairwise compared, across the biological replicates of the nine cell lines, the abundances of all the identified proteins in the immunoprecipitates with the abundances of eight important proteasome sub-complexes or regulators, that we called ‘references’. These were the 19S, PA28αβ, PA28γ, and PA200 activators, the PI31 proteasome regulator, the two major 20S proteasome subtypes, the sP20S (represented by the β5 catalytic subunit) and the iP20S (represented by the β2i catalytic subunit; Guillaume *et al*, 2010), and the ncP20S gathering the 20S non-catalytic subunits (thus representing the total 20S proteasome). The abundances of these different complexes were obtained by calculating the median PAI of their different subunits, as detailed in the Materials and Methods section. Of the 170 human proteasome-interacting proteins identified in previous AP-MS experiments (Wang & Huang, 2008; Andersen *et al*, 2009; Bousquet-Dubouch *et al*, 2009), 120 have been quantified in this survey and, among these, 70 proteins exhibited a high correlation (*R* > 0.8) with at least one of the references, suggesting that these proteins constitute reliable proteasome partners. The *R*^2^ obtained between each reference and these 70 major proteasome-associated proteins or subunits quantified in the nine cell lines proteasome preparations was used to obtain hierarchical clusters, as detailed in the Materials and Methods section, which were then represented with a heat-map (Fig4A). The 20S assembly chaperones, proteasome assembly chaperones (PACs) 1–4 and POMP, which were all detected in the AP-MS experiments from the nine cell lines, were also included in this experiment thus containing 73 proteins in total (Supplementary Table S2).

Heat-map representing the correlations (expressed as the *R*^2^) between the abundances of 73 known proteasome-interacting proteins (PIPs) and the abundances of 8 reference proteins or protein complexes, PA28γ, β2i (representing the iP20S), PA28αβ, ncP20S (median of α1–α7, β3, β4, β6, and β7 profiles), 19S (median of Rpt1–6, Rpn1–3, 5–14 profiles), PI31, β5 (representing the sP20S), and PA200. For protein complexes, the median PAI of their subunits in each of the 24 AP-MS experiments was used: α1–α7, β3, β4, β6, and β7 subunits for the ncP20S, Rpt1–6, Rpn1–3, 5–14 for the 19S RP, and PA28α and PA28β subunits for the PA28αβ RP. The *R*^2^ values were hierarchically clustered. Three distinct clusters of composition detailed hereafter could be obtained. Cluster 1 (from top to bottom): Rpt3, Rpn13, α2, Rpn7, USP14, hHR23B, α1, β6, β3, α4, α7, Rpn6, Rpn3, Rpt4, Rpn10, Rpn5, Rpt5, Rpn1, Rpn11, Rpt1, Rpn9, Rpn2, Rpn8, α3, α6, β4, Rpt6, PDC6, Rpn12, APEH, Ubiquilin-1, α5, β7, CCT7, CCT4, CCT2, CCT3, CCT5, CCT6A, DNAJA1, HSP90AB1, HSP90AA1, PNP, Rpt2. Cluster 2 (from top to bottom): 14-3-3ζ/δ, CAND1, GSR, UBE3C, β2, ATP5A1, ATP5B, β1, PA200, β5, FBXO7, UCHL5, TXNL1, ECM29, PI31. Cluster 3 (from top to bottom): PA28β, PITH1, β2i, PA28α, PA28γ, β5i, β1i.

Principal component analysis (PCA) of the abundances of 73 known PIPs. The circles represent the main clusters observed (iP20S, ncP20S/19S, sP20S and the 20S assembly chaperones).

Plot of the *R*^2^ values between the iP20S or the sP20S and 193 protein correlating (*R*^2^ > 0.8) with the iP20S, the sP20S, or the ncP20S.

Using this supervised clustering approach, three major protein clusters can be clearly highlighted. A first cluster is formed by proteins whose abundances clearly correlate with those of the ncP20S or the 19S RP references. These proteins gather all the non-catalytic 20S subunits and 19S subunits, but also proteins which are known to associate with the 19S RP-like USP14, a major deubiquitinylase, and hHR23B and Ubiquilin-1, two proteins shuttling polyubiquitinylated substrates to the 19S. A second cluster of proteins was found to correlate with the sP20S, PA200, and PI31 references. This protein group is composed of all the standard catalytic subunits (β1, β2, and β5), PI31 and PA200 regulators, but also includes the proteasome-associated proteins ECM29, Fbxo7, and UchL5. Of note, PI31 and Fbox7 have been shown to dimerize although the functional role of this interaction needs to be elucidated (Kirk *et al*, 2008). Finally, a third cluster gathers proteins correlating with the iP20S and the PA28αβ references. In this cluster are grouped all the immunocatalytic subunits (β1i, β2i, and β5i) as well as the PA28α and PA28β proteins. Strikingly, these proteins correlate very poorly with the sP20S reference (*R*^2^ < 0.1), and moderately or poorly with the ncP20S and the 19S (0.1 < *R*^2^ < 0.6). PA28γ settles in this 3^rd^ cluster although it moderately correlates with the iP20S (*R*^2^ = 0.66) and with PA28αβ (*R*^2^ = 0.67).

To confirm these results, we used two complementary and unsupervised statistical methods, the principal component analysis (PCA) and the agglomerative hierarchical clustering (AHC), where no reference proteins or protein groups were used to correlate the abundances. Using both approaches, ncP20S, iP20S, and sP20S are again found in three very distinct protein groups and cluster with the same partners as those found with the supervised clustering method (Fig4B and Supplementary Fig S3A). Using the AHC, PA28γ is found in a different group as the iP20S and the PA28αβ RP, which are still found in the same cluster. Interestingly, UBE3C and UCHL5, two proteasome-interacting proteins (PIPs) of antagonizing ubiquitinating and deubiquitinating activities, respectively, are found in the same cluster. The PCA also appeared to efficiently cluster the group of proteasome assembly chaperones (PACs 1–4), which could not be correlated with any of the references chosen in the supervised hierarchical clustering statistical method. This might be explained by the fact that these proteins interact with 20S proteasome assembly intermediates and not with matured proteasome forms. As these chaperones cluster with none of the 20S proteasome types, our results suggest that none of the five 20S assembly chaperones is specifically involved in the formation of a particular 20S proteasome form.

Altogether, these results strongly suggest for the first time that the associations between the proteasome 20S subtypes (ncP20S representing total 20S proteasome, sP20S, iP20S), regulators (19S, PA28αβ, PA28γ, PA200, PI31), and associated proteins (ubiquitinating and deubiquitinating enzymes, Ecm29, shuttling factors) do not occur randomly and that preferential interactions exist within proteasome complexes. Coupling AP-MS to protein correlation profiling also allowed to more comprehensively characterize proteasome heterogeneity because a much higher number of known proteasome-interacting proteins (PIPs) were identified (120 PIPs) as compared to the initial glycerol gradient analysis (where 73 PIPs were identified).

### Standard and immunoproteasome interact with a different subset of proteins

An unexpected result emphasized by the clustering methods presented above is that the two main 20S proteasome subtypes, the sP20S and the iP20S (represented by the β5 and β2i catalytic subunits, respectively), partition in very different groups of proteins, whatever the statistical approach used (Fig4A and B; Supplementary Fig S2A). This result therefore suggests that sP20S and iP20S interact with a different subset of proteins, in particular with distinct regulators. In proteasome immunoprecipitates, the β5 subunit is indeed highly correlated with PI31 and PA200 (*R*^2^ of 0.89 and 0.87, respectively) but not with the PA28αβ and PA28γ complexes (*R*^2^ of 3·10^−5^ and 0.06, respectively) (Supplementary Fig S2B). On the other hand, the β2i subunit correlated well with the 20S proteasome-associated PA28αβ complex (*R*^2^ of 0.86) but very badly with PA200 and PI31 (*R*^2^ of 0.01 and 0.03, respectively) (Supplementary Fig S2B). Conversely, the 19S regulator does not correlate with any of the two 20S proteasome subtypes as it exhibits almost identical coefficients of determination for both the sP20S (*R*^2^ of 0.49) and the iP20S (*R*^2^ of 0.44) (Supplementary Fig S2B). Its abundance is rather correlated with the one of the ncP20S (*R*^2^ of 0.93), which represents all 20S proteasome forms (Supplementary Fig S2B). This means that the 19S RP associates as well with the sP20S as with the iP20S.

As far as the origin of the cell lines is considered, the three lymphoid and myeloid cell lines (U937, KG1a, and NB4) display, as expected, the highest amounts of immunoproteasome (the β2i subunit corresponds to 10–30% of the total P20S) and P20S-associated PA28αβ activator (Supplementary Fig S3C), and the lowest fractions of standard proteasome (20–50% of the total P20S) (Supplementary Fig S3A and B) and P20S-associated PA200 and PI31 regulators, compared to the other cell lines.

Besides, expression controls show that the differential interactions observed within proteasomes seem not be caused by higher or lower expressions of the subunits forming these complexes (Supplementary Fig S4 and Supplementary Table S3). To check more accurately this point, the expression levels of proteasome and RPs subunits in the different cell lines were plotted (Supplementary Fig S5A), as previously performed for subunits abundances measured in proteasome immunoprecipitates (Supplementary Fig S2B and Supplementary Table S3). Noteworthy, some of the correlations measured in purified proteasomes were conserved in total cell lysates (for instance, the good correlation between the 20S proteasome α6 and α7 subunits, *R*^2^ = 0.8, or the one between PA28α and PA28β, *R*^2^ = 0.8). However, many other subunits expression levels could not be correlated, such as the ones among 19S subunits (Supplementary Figs S1D and S5A) or, more importantly, the ones between β2i and PA28αβ (*R*^2^ = 0.37) and between β5 and PI31 (*R*^2^ = 0.15) (Supplementary Fig S5A). PA200's expression level is, however, correlated with the one of β5 (*R*^2^ = 0.82).

Next, to widen our correlation data to a larger group of putative proteasome-interacting proteins (PIPs), we correlated the abundances of sP20S and iP20S with 193 proteins found confidently associated with the purified proteasome, as these proteins exhibit high correlation (*R* > 0.8) with at least one of the three 20S CP subtypes (total proteasome (ncP20S), sP20S, and iP20S). These proteins could be quantified with at least two peptides and display median signal-to-noise ratios above 10 (Supplementary Table S4). Figure4C shows the *R*^2^ obtained between all these proteins and the sP20S or the iP20S. Strikingly, the curves appear inverted, showing that proteins whose presence highly correlates with the sP20S are not found associated with the iP20S, and vice versa. Importantly, no such trend was observed in the total lysates of the nine cell lines (at least for the 186 out of 193 putative PIPs which could be identified; Supplementary Table S4 and Supplementary Fig S5B). In total, 60 putative PIPs correlate (*R* > 0.8) with the sP20S and 31 do with the iP20S in the proteasome immunoprecipitates (Fig4C). These proteins therefore constitute possible substrates or PIPs associating preferentially with one particular 20S proteasome subtype.

**Figure 5:**
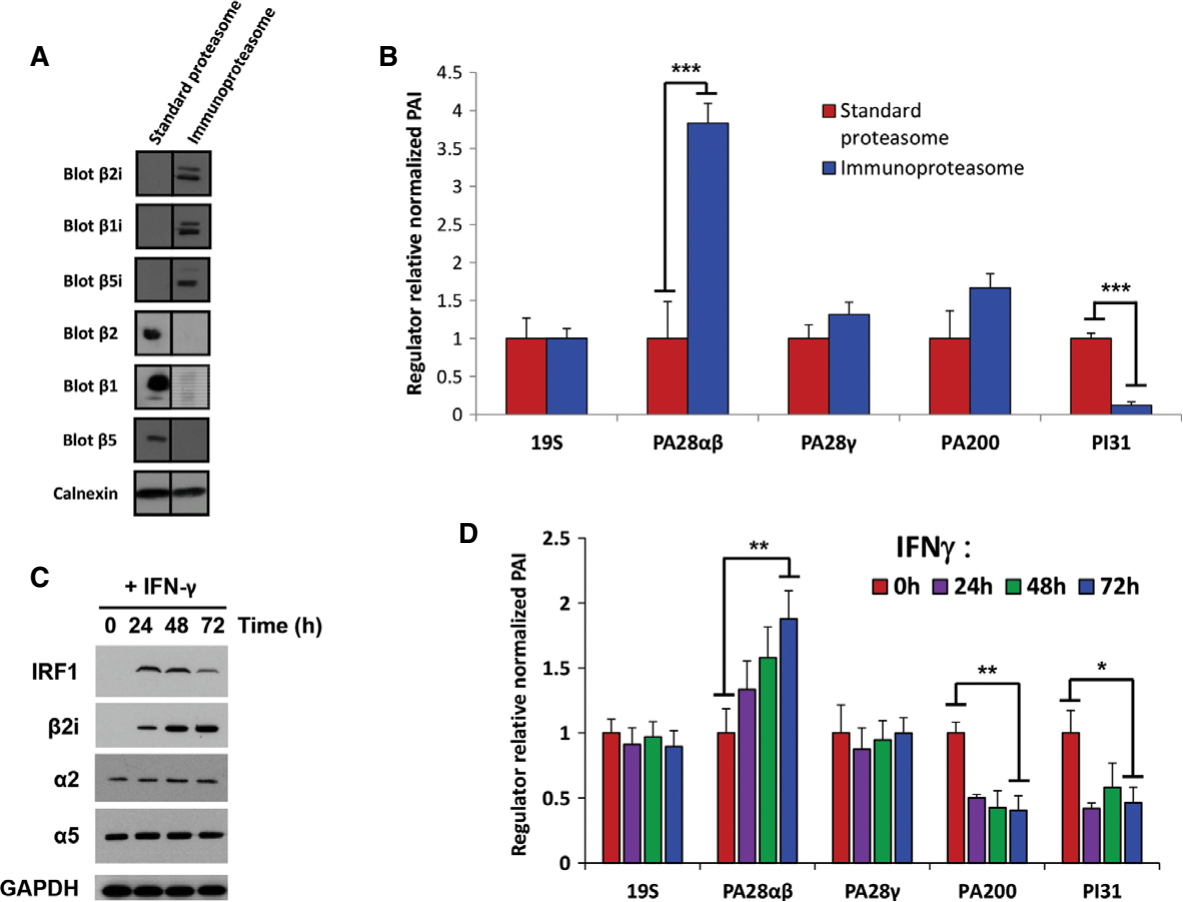
Changes in the expression of 20S proteasome catalytic subunits modulate 20S-associated regulators

To further confirm the preferential association of sP20S and iP20S with a different subset of regulators, we next used two cell lines expressing a unique form of these two 20S subtypes. Proteasomes were affinity-purified from formaldehyde cross-linked HEK EBNA cells, either untransfected (containing mainly sP20S) or transfected with the three immunocatalytic subunits β5i, β1i and β2i, so that these cells contain only iP20S (Fig5A). Precipitates were analyzed by nano-LC-MS/MS and quantified by the TOP3 label-free quantitative method (Supplementary Table S5). In each sample, for a given regulator (19S, PA28αβ, PA28γ, PA200, and PI31), the protein abundance index was defined as the mean of the PAIs of all the proteins belonging to this regulator and was normalized by the PAI of the ncP20S, to obtain a normalized PAI. Then, the regulator's averaged (*n* = 4 biological replicates) normalized PAIs were set to 1 for the cell lines containing the sP20S to obtain a regulator relative normalized PAI. As shown in Fig5B, using these two model cell lines, we observe that the 19S, PA28γ, and PA200 associate equally with the sP20S and the iP20S. On the other hand, the interaction of PA28αβ with the iP20S is nearly fourfold higher than with the sP20S, and the relative abundance of PI31 is increased by a factor superior to 8 in the sP20S immunoprecipitate when compared to that of iP20S. Interestingly, proteasome immunopurified from HEK EBNA cells transfected with either β5i alone or with β1i and β5i (Supplementary Fig S6A) (and therefore expressing a unique intermediate P20S, β5i intermediate 20S proteasome (β5i P20S) or β1iβ5i intermediate 20S proteasome (β1iβ5i P20S), respectively (Guillaume *et al*, 2010)) associate with the same main P20S proteasome regulators as the immunoproteasome (Supplementary Fig S6B). The preferential association of PA28αβ with the iP20S was further confirmed by a Western blot analysis using another system of overexpression of the immunocatalytic subunits (HEK T-Rex) (Supplementary Fig S6C). Although PA28α and PA28β proteins were equally abundant in the two cell lysates containing either the β5 subunit (representing the sP20S) or the β2i subunit (representing the iP20S), the MCP21 immunoprecipitate of the iP20S showed a much higher quantity of co-immunoprecipitated PA28α and PA28β proteins than the MCP21 immunoprecipitate of sP20S.

The two HEK EBNA cell lines express only standard proteasome or immunoproteasome subunits. Western blots against the immuno- (β1i, β2i, β5i) and standard (β1, β2, β5) catalytic subunits of the 20S proteasome. Calnexin is used as a loading control. Black lines delineate the boundary between vertically sliced images that juxtapose lanes that were non-adjacent in the gel. Importantly, the bands were assembled from the same blot.

Relative normalized abundance indexes of proteasome regulators in HEK EBNA cells containing only immunoproteasome compared to HEK EBNA cells containing only standard proteasome. The normalized abundance indexes for each regulator were set to 1 for standard proteasome conditions (*n *=* *4).

Kinetics of IFN-γ treatment on HeLa cells. HeLa cells were stimulated for 0, 24, 48, or 72 h with IFN-γ. Western blots were performed on total cell lysates with antibodies against the β2i, α2, and α5 subunits. IRF-1 was used to control IFN-γ treatment efficiency, and GAPDH was used as a loading control.

For each time point of the IFN-γ treatment, proteasome complexes were purified and analyzed by LC-MS/MS. Proteasome complexes dynamics was measured by label-free quantitative proteomics. The normalized abundance index of each protein or protein complex obtained at each time point was compared to the one obtained at the 0 h time point to obtain a regulator relative normalized PAI (*n *=* *3). Data information: *P <* *0.05; **P <* *0.01; ***P <* *0.001 (Student *t*-test). Source data are available online for this figure

Finally, we took advantage of the ability of IFNγ to physiologically change the relative proportions of sP20S and iP20S to validate further the preferential association of sP20S and iP20S with a different subset of regulators. HeLa cells, which contain a low level of iP20S at basal state (Fabre *et al*, 2014a,b), were treated with IFNγ during 72 h. IRF1, the transcription factor responsible for the expression of the catalytic immunosubunits, was rapidly induced (Fig5C), which leads to a strong increase in the expression of all the catalytic immunosubunits, and especially of β2i, together with a decrease of the incorporation of the three standard catalytic subunits in the proteasome complexes (Supplementary Fig S7A). More precisely, the quantitative results show an eightfold increase of iP20S and a twofold decrease of sP20S, while no significant change of the total 20S proteasome abundance was observed (no significant variations of the α2 and α5 subunits, Fig5C). Next, we quantified the dynamics of 20S-associated 19S, PA28αβ, PA28γ, PA200, and PI31 regulators under IFNγ treatment, by analyzing the abundances of these proteasome-associated proteins in immunopurified proteasomes at each time point, as explained earlier (Fig5D andSupplementary Table S6). No significant variations of 19S and PA28γ were noticed while the trends of PA28αβ on one hand and of PA200 and PI31 on the other hand followed the ones of the iP20S and of the sP20S, respectively. These differential interactions, in particular the ones involving PI31 and PA200, are not caused by changes of the expression of these proteins in the total lysates of the IFNγ-stimulated HeLa cells (Supplementary Fig S7B and Supplementary Table S7). These results thus strongly suggest that, in a physiological context where significant variations of the sP20S and iP20S abundances are observed, the quantities of some of the 20S-associated regulators fluctuate in parallel to the quantities of sP20S or iP20S. Noteworthy, PA200's abundance is correlated with that of the sP20S both in the nine cell lines and in the IFNγ-stimulated HeLa cells, but not in the HEK EBNA systems, which, however, constitutes a less physiologically relevant model. Altogether, these results clearly show for the first time a preferential interaction of a subset of 20S proteasome regulators, in particular PA28αβ, PA200, and PI31, with either the sP20S or the iP20S.

Recently, the in-depth proteomic profiling of human proteins from tissue or cell lines samples from diverse origins was published (Kim *et al*, 2014; Wilhelm *et al*, 2014). From the web-based resource made freely available by Kim *et al* (Kim *et al*, 2014), we could extract the heat-map representing the abundances of sP20S, iP20S, PA28αβ RP, PA200 RP, and PI31 in 30 histologically normal samples from diverse tissues, either fetal or adult (Supplementary Fig S8). As expected, iP20S and PA28αβ are clearly overexpressed in antigen-presenting cells. Strikingly, PI31's expression is well correlated with the abundance of sP20S, in particular in all the fetal tissues studied. These results suggest a possible role of the association of PI31 with the sP20S in fetal development. Interestingly, the expression of PA200 is high and matches well that of sP20S in adult reproductive tissues, but not in fetal ones. This result thus correlates with a known important function of PA200 during spermatogenesis (Khor *et al*, 2006). However, as demonstrated in this study using AP-MS, correlated levels of expression between two protein complex components do not necessarily involve their common integration within a complex.

## Discussion

Proteasomes constitute dynamic structures with respect to the cellular environment, the cell type, the subcellular localization, the tissue, or in response to physiological and external perturbations. They adapt to these diverse biological contexts through changing their overall subunit composition and association with diverse regulatory particles (RPs) and proteasome-interacting proteins (PIPs). Proteasome diversity could thus reflect specialized functions of each individual proteasome form. To address proteasome heterogeneity, we set out to resolve the various complexes in which the different 20S CPs might be involved in. To support more generalized conclusions, we used a large variety of human cell lines exhibiting different proteasome compositions, as well as complementary methodological approaches combining the sensitivity of AP-MS and the resolving power of PCP-MS.

AP-MS is a global strategy that has efficiently revealed hundreds of proteasome-associated proteins (PIPs) (Bousquet-Dubouch *et al*, 2011; Kaake *et al*, 2014) but that did not assign them to particular 20S proteasome subtypes. Alternatively, protein correlation profiling associated with MS can distinguish different related sub-complexes. A given protein might be assigned to a given complex through the similarity of protein profiles across chromatographic fractions. Recently, this approach has proven to be efficient for the determination of the quantitative distribution of the 19S RP among the singly and doubly capped proteasome complexes using a high-resolution SEC approach (Kristensen *et al*, 2012). In this work, 300 protein complexes were identified from HeLa cells lysates using stringent peak features computational filtering. However, given the high dynamic range of mammalian cells proteins, only the most abundant and stable subunits of a given protein complex might be identified and confidently clustered. Havugimana *et al* (2012) doubled the depth of the analysis (622 putative protein complexes identified) but at the cost of a high fractionation effort (more than 1,000 fractions analyzed). The PCP-MS analysis we performed on U937 AML cells proteins separated by glycerol density gradient ultracentrifugation enabled to reveal for the first time a previously unreported preferential association between iP20S and PA28αβ RP. However, in the 19 glycerol gradient fractions analyzed, only the most abundant proteasome regulators such as 19S, PA28αβ, or PA28γ could be identified, probably because of the low fractionation level used. Many of the other known PIPs were missing, probably because of their sub-stoichiometric integration into proteasomes. So, as an alternative to extensive fractionation, we developed a new workflow combining the strengths of AP-MS and PCP-MS to significantly increase the number of PIPs identified and thus to comprehensively analyze proteasome complexes heterogeneity.

Contrary to all previous surveys where PCP-MS was applied on elution profiles resulting from biochemical fractionation of protein complexes arising from a unique biological sample, we used a new strategy where the quantitative MS abundances of proteasome subunits and associated proteins were compared across 24 affinity-purified proteasome samples obtained from a wide range of nine human cell lines exhibiting very diverse proteasome composition (Fabre *et al*, 2014a,b). Although this approach is targeted to a protein complex of interest and thus low throughput as compared to the SEC-based PCP-MS strategy, it allows going deeper into the characterization of a heterogeneous protein complex such as the proteasome. As previously reported using PCP-MS on chromatographic elution profiles (Kristensen *et al*, 2012; Kirkwood *et al*, 2013), we assumed that two proteins that are always present together in the same complex have similar abundance patterns in the affinity-purified samples. Using independent supervised and unsupervised statistical approaches, hierarchical clustering and principal component analysis, respectively, proteasome subunits and interacting proteins were clustered according to their abundances in the 24 proteasome immunoprecipitates. Interestingly, core subunits of known proteasome sub-complexes, such as the 19S or the PA28αβ RPs, expectedly partition into the same group, which emphasizes the quality of the data but also the relevance of the statistical approaches.

Two main forms of functional 20S proteasomes are present in higher eukaryotes, the standard proteasome (sP20S) and the immunoproteasome (iP20S), that differ by their three beta catalytic subunits and that share the seven alpha and the four other beta subunits. We arbitrarily called in this study 20S non-catalytic proteasome (ncP20S) as the subset of the 11 common proteins (α1–α7, β3, β4, β6, and β7), which are present in all 20S proteasome forms; ncP20S thus represents the total 20S core particle. A protein that will be associated with the two functional 20S proteasome forms will correlate with subunits of the ncP20S, while a protein that will be preferentially associated with the sP20S or with the iP20S will correlate with the β5 subunit or with the β2i subunit, respectively. An unexpected and striking result arising from the statistical analysis of the data was that the 20S subunits can be clustered into three different groups in which the ncP20S, the iP20S, and the sP20S clearly emerge as favorite partners of distinct subsets of proteins, and in particular to different important 20S CP regulators, such as the 19S RP, the PA28αβ RP, and the PA200 and PI31 proteins, respectively. These preferential associations were further confirmed by changing the relative proportions of the two 20S CP subtypes, either using model cell lines containing a unique subtype of 20S proteasome, or physiologically, by IFNγ stimulation.

Altogether, our data demonstrate, through the different approaches used, that the 19S RP does not have any preference to any of the two sP20S and iP20S subtypes. This probably emphasizes the ubiquitous role of the 19S RP, a very broadly conserved proteasome activator (Stadtmueller & Hill, 2011) which is indeed the only proteasome-associated regulator required for the ubiquitin-dependent protein degradation pathway, a system involved in all major cellular processes of eukaryotic organisms.

On the contrary, PA28α and PA28β subunits are clustered with the 3 beta catalytic immunosubunits. The iP20S associated with the PA28αβ RP thus represents a functional subclass of proteasome complex. The two subunits constituting the PA28αβ RP are IFNγ inducible, and, consistent with this property, many studies have implicated PA28αβ in the efficient production of MHC class I ligands both *in vitro* and *in vivo* (Dick *et al*, 1996; Groettrup *et al*, 1996, 2010; Schwarz *et al*, 2000). However, the mechanistic basis for this function remains elusive. The primary actor of IFNγ-induced epitope generation is the iP20S because its cleavage specificity is different from the one of the sP20S and may favor the generation of antigenic peptides sequences which can be loaded on MHC class I molecules (Romero *et al*, 1991; Toes *et al*, 2001; Basler *et al*, 2013). The preferred interaction we observed between iP20S and PA28αβ might at least in part explain the positive effect of PA28αβ on the CTL response, in particular in the early IFNγ-stimulating phase, when newly assembled iP20S is not abundant. Indeed, the preferential incorporation of β1i, β2i, and β5i inducible subunits into newly assembled 20S proteasome takes time, at least 8–12 h (Seifert *et al*, 2010), and the ability of PA28αβ to modulate the quantity but also the quality of peptide repertoire produced by the 20S proteasome (Dick *et al*, 1996; Shimbara *et al*, 1997; Cascio, 2014; Raule *et al*, 2014) might therefore trigger an effective and rapid CTL response. How the binding of PA28αβ to the 20S proteasome could affect proteasome activity and cleavage specificity was recently studied by in solution NMR spectroscopic analysis of the archaeal *T. acidophilum* 20S proteasome bound to the 11S RP (PA26) (Ruschak & Kay, 2012). CP catalytic proteolysis was indeed shown to be controlled and modulated through allosteric mechanisms by which 11S binding is communicated to active sites distant of about 75 Å. Another biological role that is common to both iP20S and PA28αβ (Seifert *et al*, 2010) resides in their general protective function from oxidative stress in several biological contexts (Pickering *et al*, 2010; Seifert *et al*, 2010; Li *et al*, 2011a,b; Hernebring *et al*, 2013). Our findings concerning the preferred association of iP20S and PA28αβ could explain the observed enhanced activity of iP20S to clear the defective ribosomal products generated during IFN-induced oxidative stress (Seifert *et al*, 2010) and, more generally, could account for their common role in the maintenance of protein homeostasis in oxidant conditions.

Our results strongly suggest that PI31 associates preferentially with sP20S and much less with iP20S in a wide range of human cell lines. PI31 was first described as a natural 20S proteasome inhibitor *in vitro* (McCutchen-Maloney *et al*, 2000), but this function was not confirmed *in vivo* (Zaiss *et al*, 2002) and the regulatory role of PI31 on the proteasome still remains unclear (Li *et al*, 2014). Whether a functional link between sP20S and PI31 would account for the repression of IFNγ-induced iP20S maturation exerted by PI31 (Zaiss *et al*, 2002) could be the subject of further investigation.

Another important consideration to be addressed is the structural basis of the preferential association of sP20S and iP20S with a different subset of 20S regulators. Recent biochemical studies have demonstrated that modification at the active sites leads to long distance gate opening (Osmulski *et al*, 2009) and to changes in binding affinities for RPs (Kleijnen *et al*, 2007). Therefore, this suggests that the replacement of the β1, β2, and β5 subunits of the sP20S by their immunocounterparts β1i, β2i, and β5i in the iP20S could modulate RPs binding. The allosteric effect described by Ruschak and Kay (2012) might well be operative in more complex CPs because of the high interspecies conservation of CPs mechanism of proteolysis and RPs binding (Stadtmueller & Hill, 2011). We therefore believe that the structural differences at the active sites of human sP20S and iP20S might well account for the fact that they interact with a different subset of proteins, and more precisely with different RPs such as PA28αβ, PA200 or PI31. Noteworthy, PA200 and Ecm29 are both large monomeric proteins that interact with the α rings via their HEAT repeat domain (Savulescu & Glickman, 2011). This structural similarity might explain their common preferred association with the sP20S subtype.

Besides 73 well-characterized proteins of the proteasome complexes, this study allowed to identify 123 additional proteins whose abundance correlated with one of the two major 20S CPs, sP20S and iP20S, or with the ncP20S. Unexpectedly, they could also be clustered in three groups equally distributed between iP20S and sP20S, or preferentially associated with one of the two. This unequal distribution suggests that these proteins are not contaminants of the immunopurified proteasome fractions but are functional players or proteasome substrates.

More generally, the presented strategy could be widened to unravel the dynamic and heterogeneous nature of many other biologically relevant molecular systems.

## Materials and Methods

### Cell lines and culture conditions

HEK 293T, HCT116, RKO, and U2OS cell lines were grown in DMEM media supplemented with 10% fetal bovine serum (FBS). U937, HeLa S3, and NB4 cell lines were grown in RPMI 1640 media supplemented with 10% FBS. KG1a cell line was grown in RPMI 1640 media supplemented with 20% FBS. MRC5 cell line was grown in MEM-α media supplemented with 10% FBS. All cell lines were cultured with 2 × 10^−3^ M glutamine, 100 Units/ml penicillin, 100 μg/ml streptomycin at 37°C, and 5% CO_2_. Unsynchronized cells were harvested at 80% of confluence for adherent cells or at a concentration of 1 × 10^6^ cells per ml of culture for suspension cells. HEK EBNA and HEK T-Rex cells were grown as described elsewhere (Chapiro *et al*, 2006; Guillaume *et al*, 2010). HeLa cells were treated with interferon-γ (R&D Systems, Minneapolis, MN, USA) at a concentration of 100 ng/ml in fresh medium.

### Formaldehyde *in vivo* cross-linking, and proteasome purification and quantification

Formaldehyde *in vivo* cross-linking was performed with a concentration of 0.1% at 37°C during 15 min. The cross-linking reaction was quenched with addition of 125 mM of glycine, and cells were washed three times with PBS and stored at −80°C. Cells were lysed with 2 ml of lysis buffer (10 mM Hepes pH 7.9, 10 mM KCl, 5 mM MgCl_2_, 10% glycerol, 10 mM ATP, 1% NP-40, protease and phosphatase inhibitor; Roche) for 15 min at 4°C, sonicated, and centrifuged. Protein concentration was determined by detergent-compatible assay (DC assay; Bio-Rad). 20S Proteasome purification and quantification by sandwich ELISA assay were performed as previously described (Fabre *et al*, 2013). Briefly, for 20S proteasome purification, each cell lysate sample was incubated with 100 mg of CNBr sepharose beads (GE Healthcare) coupled with 0.8 mg MCP21 antibody (directed against the α2 subunit of the 20S proteasome). Supernatants (S1) were collected, and beads were washed three times with 40 bead volumes of washing buffer (20 mM Tris–HCl pH 8, 1 mM EDTA, 10% glycerol, 150 mM NaCl, 0.1% NP-40, 10 mM ATP and 2 mM MgCl_2_), and proteins were eluted with 0.5 ml of elution buffer (20 mM Tris–HCl pH 8, 1 mM EDTA, 10% glycerol, 3 M NaCl, 10 mM ATP and 2 mM MgCl_2_). Beads were then washed and incubated with supernatants S1. Two additional cycles of purification were repeated, and the three eluates were finally pooled together.

### Fractionation of proteasome complexes by glycerol gradient sedimentation

2 × 10^8^ U937 cross-linked cells were lysed with 2 ml of lysis buffer and centrifuged at 14,000 *g* for 10 min. Samples were concentrated to a final volume of 500 μl using an ultrafiltration device with a cutoff of 100 kDa (Millipore). Samples were then fractionated by 15–40% (v/v) (100 mM Tris–HCl pH 7.4, 0.15 M NaCl, 0.5 M MgCl_2_, 2 mM ATP, 15–40% glycerol) linear glycerol density gradient ultra-centrifugation (22 h; 96,500 *g*) using a Beckman SW 28 rotor. The gradient was separated into 32 fractions of 1 ml. Proteasome activity was measured in each fraction as previously described (Fabre *et al*, 2013). The 19 fractions (corresponding to glycerol percentages of 24–40%) where proteasomal activity was detected were used for the LC-MS/MS and PCP analyses. 1% of each glycerol gradient fraction was used for LC-MS/MS analysis. Before injection on the nano-LC, eight isotopically labeled peptides (AQUA peptides) eluting all along the chromatographic gradient were spiked (100 fmol of each peptide per injection) to normalize the MS inter-run signal intensity variations.

### Detailed LC-MS/MS analysis, data search, and validation

Each purified proteasome sample or glycerol gradient fraction was precipitated with 20% TCA and washed with acetone. Samples were boiled 30 min at 95°C in Laemmli buffer to denature proteins and reverse formaldehyde cross-link, as previously optimized (Fabre *et al*, 2013). Proteins were alkylated with 100 mM chloro-acetamide for 30 min at room temperature in the dark. Proteins were concentrated in a single band on a 12% acrylamide SDS–PAGE gel and visualized by colloidal Coomassie Blue staining. One-shot analysis of the entire mixture was performed. A single band, containing the whole sample, was cut and washed in 50 mM ammonium bicarbonate for 15 min at 37°C followed by a second wash in 50 mM ammonium bicarbonate, acetonitrile (1:1) for 15 min at 37°C, and a final dehydration in 100% ACN. Trypsin (Promega) digestion was performed over night at 37°C. The resulting peptides were extracted from the gel by three steps: a first incubation in 50 mM ammonium bicarbonate for 15 min at 37°C and two incubations in 10% formic acid, acetonitrile (1:1) for 15 min at 37°C. The three collected extractions were pooled with the initial digestion supernatant, dried in a Speed-Vac, and resuspended with 2% acetonitrile, 0.05% trifluoroacetic acid. The peptides mixtures were analyzed by nano-LC-MS/MS using an UltiMate 3000 system (Dionex) coupled to LTQ-Orbitrap XL or Velos mass spectrometers (Thermo Fisher Scientific, Bremen, Germany). Five microliters of each peptide sample corresponding to an equivalent initial quantity of 20S proteasome (estimated by Elisa) of 2.5 μg for purified proteasome samples or 5 μg of total proteins for the glycerol gradient fractions were loaded on a C18 precolumn (300 μm inner diameter × 5 mm; Dionex) at 20 μl/min in 5% acetonitrile, 0.05% trifluoroacetic acid. After 5 min of desalting, the precolumn was switched online with the analytical C18 column (75 μm inner diameter × 15 cm; PepMap C18, Dionex) equilibrated in 95% solvent A (5% acetonitrile, 0.2% formic acid) and 5% solvent B (80% acetonitrile, 0.2% formic acid). Peptides were eluted using a 5–50% gradient of solvent B during 160 min at a 300 nl/min flow rate. The LTQ-Orbitrap XL was operated in data-dependent acquisition mode with the Xcalibur software. Survey scan MS spectra were acquired in the Orbitrap on the 350–1,800 *m*/*z* range with the resolution set to a value of 60,000. The five (LTQ-Orbitrap XL) or twenty (LTQ-Orbitrap Velos) most intense ions per survey scan were selected for CID fragmentation, and the resulting fragments were analyzed in the linear trap (LTQ). Dynamic exclusion was used within 60 s to prevent repetitive selection of the same peptide. The Mascot Daemon software (version 2.3.2; Matrix Science, London, UK) was used to perform database searches, using the Extract_msn.exe macro provided with Xcalibur (version 2.0 SR2; Thermo Fisher Scientific) to generate peaklists. The following parameters were set for creation of the peaklists: parent ions in the mass range 400–4,500, no grouping of MS/MS scans, and threshold at 1,000. A peaklist was created for each analyzed fraction, and individual Mascot (version 2.3.01) searches were performed for each fraction. The mass tolerances in MS and MS/MS were set to 5 ppm and 0.8 Da, respectively, and the instrument setting was specified as ‘ESI-TRAP’. Trypsin was designated as the protease (specificity set for cleavage after Lys or Arg), and up to two missed cleavages were allowed. Oxidation of methionine and amino-terminal protein acetylation were searched as variable modifications. Carbamidomethylation on cysteine was set as fixed modification. Protein hits were automatically validated with a false discovery rate (FDR) of 1% on proteins and 5% on peptides (minimum peptide length of 6 amino acids). To evaluate false-positive rates, all the initial database searches were performed using the ‘decoy’ option of Mascot, that is, the data were searched against a combined database containing the real specified protein sequences (target database, Swiss-Prot human, release 2013_01, 20,232 entries) and the corresponding reversed protein sequences (decoy database). Mascot file parsing and quantification (MFPaQ) used the same criteria to validate decoy and target hits, calculated the false discovery rate [FDR = number of validated decoy hits/(number of validated target hits + number of validated decoy hits) × 100]. Proteins identified with exactly the same set of peptides were grouped, and only one member of the protein group was reported (the one that we considered as the most significant according to the functional description given in the UniProt Knowledgebase). Highly homologous protein hits, that is, proteins identified with top ranking MS/MS queries also assigned to another protein hit of higher score (red, non-bold peptides), were detected by the MFPaQ software (Mouton-Barbosa *et al*, 2010) and were considered as individual hits and included in the final list only if they were additionally assigned a specific top ranking (red and bold) peptide of score higher than 30 (*P*-value < 0.05).

### Data quantification

#### Relative quantification of proteins

Quantification of proteins was performed using the label-free module implemented in the MFPaQ v4.0.0 software (http://mfpaq.sourceforge.net/) (Bouyssie *et al*, 2007; Mouton-Barbosa *et al*, 2010; Gautier *et al*, 2012). For each sample, the software uses the validated identification results and extracts ion chromatograms (XIC) of the identified peptide ions in the corresponding raw nano-LC-MS files, based on their experimentally measured retention time (RT) and monoisotopic *m*/*z* values. The time value used for this process is retrieved from Mascot result files, based on an MS2 event matching to the peptide ion. If several MS2 events were matched to a given peptide ion, the software checks the intensity of each corresponding precursor peak in the previous MS survey scan. The time of the MS scan, which exhibits the highest precursor ion intensity, is attributed to the peptide ion and then used for XIC extraction as well as for the alignment process. Peptide ions identified in all the samples to be compared were used to build a retention time matrix in order to align LC-MS runs. If some peptide ions were sequenced by MS/MS and validated only in some of the samples to be compared, their XIC signal was extracted in the nano-LC-MS raw file of the other samples using a predicted RT value calculated from this alignment matrix by a linear interpolation method. Quantification of peptide ions was performed based on calculated XIC areas values. Only peptides with a Mascot score higher than 30 (*P*-value < 0.05) at least in one of the samples were selected for the quantification. In order to perform protein relative quantification in different samples, a protein abundance index (PAI) was calculated. It is defined as the average of XIC area values for the most three intense reference tryptic peptides identified for this protein (the three peptides exhibiting the highest intensities across the different samples were selected as reference peptides, and these same three peptides were used to compute the PAI of the protein in each sample; if only one or two peptides were identified and quantified in the case of low-abundant proteins, the PAI was calculated based on their XIC area values).

### Protein correlation profiling analysis

Glycerol sedimentation profiles were normalized by dividing the protein abundance index of each protein in each fraction of the gradient by the sum of the protein abundance indexes measured in all the gradient fractions. χ^2^ values were calculated between the profiles of all the proteins identified and quantified in the glycerol gradient fractions and the reference proteins profiles with the formula 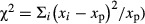 in which *i* is the fraction number, *x*_*i*_ is the normalized value in fraction *i*, and *x*_p_ is the value of the reference protein in fraction *i*, as described previously (Wiese *et al*, 2007). The sedimentation profiles of the median of the non-catalytic 20S proteasome subunits (α1–α7, β3, β4, β6 and β7), the median of 19S subunits, and the median of the PA28αβ were used as reference profiles. The χ^2^ values of the two biological replicates were plotted for each chosen reference.

### Supervised and unsupervised clustering of proteins abundances across 24 affinity-purified proteasomes samples

The supervised clustering was performed based on the *R*^2^ values (Coefficient of determination) obtained from the pairwise comparison of the abundances (PAIs), across the 24 proteasome purifications, of proteasome subunits or known interacting proteins with the abundances (PAIs) of eight reference proteasome sub-complexes or regulators. The PAIs of these eight references were calculated as follows:

- PAI of the 20S non-catalytic proteasome (ncP20S): median PAIs of subunits α1–α7, β3, β4, β6 and β7
- PAI of the standard proteasome (sP20S): PAI of subunit β5
- PAI of the immunoproteasome (iP20S): PAI of subunit β2i
- PAI of the 19S RP: median PAIs of subunits Rpt_1–6_ and Rpn_1,2,3,5,6,7,8,9,10,11,12,13_
- PAI of the PA28αβ RP: median PAIs of subunits PA28α and PA28β
- PA28γ, PA200, and PI31 are formed by a unique polypeptide chain, so their PAIs were directly used.

The heat-map was created using the R package software (version 3.1.0) and the graphic package ggplot2 (version 0.9.3.1).

The two unsupervised clustering analyses (principal component analysis, PCA, and agglomerative hierarchical clustering, AHC) were performed from normalized PAIs, across the 24 proteasome purifications, of proteasome subunits or known interacting proteins using the XLSTAT software (version 2012.3.01). The PAIs were normalized by setting the highest PAI measured for a given protein in the proteasome purifications to one. The PCA was of Pearson (n) type. For the AHC, the Pearson correlation coefficient was used to search for similarities using an unweighted pair-group average agglomeration method.

### Data availability

The mass spectrometry proteomics data have been deposited to the ProteomeXchange Consortium (Vizcaino *et al*, 2014) via the PRIDE partner repository with the dataset identifier PXD001043 (Supplementary Table S8).

## Supporting Information

Supplementary Materials and Methods

Review Process File
