# Supplementary material for: Deciphering preferential interactions within supramolecular protein complexes: the proteasome case
Source: Mol Syst Biol. 2015 Jan 5;11(1):771. doi: 10.15252/msb.20145497 (PMC4332148; doi:10.15252/msb.20145497)
Supplement: Supplementary file 1 [file msb0011-0771-sd1.pdf]

# Figure S1

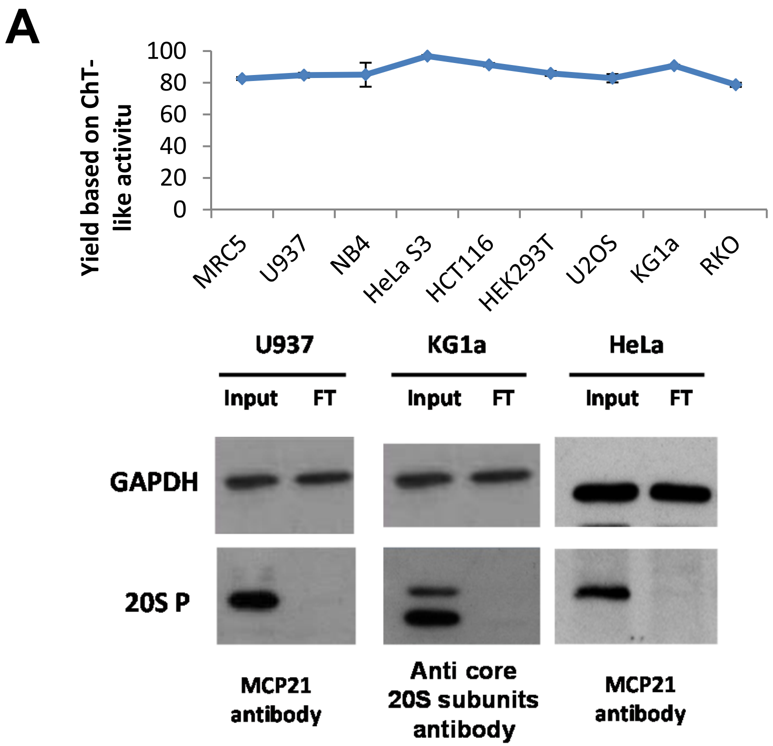


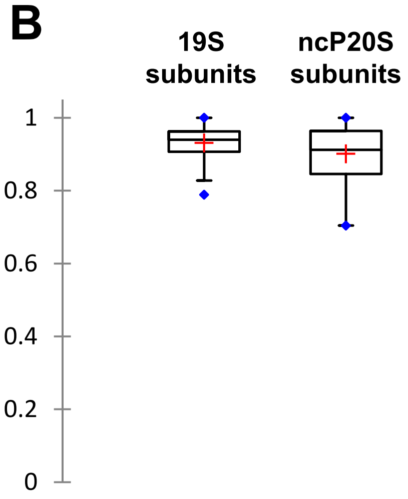


| Statistics for $R^2$ | 19S subunits | 20S subunits |
|---|---|---|
| No. of observations | 324 | 122 |
| Minimum | 0.79 | 0.70 |
| Maximum | 1.00 | 1.00 |
| 1st Quartile | 0.91 | 0.85 |
| Median | 0.94 | 0.91 |
| 3rd Quartile | 0.96 | 0.96 |
| Mean | 0.93 | 0.90 |
| Variance (n-1) | 0.00 | 0.01 |
| Standard deviation (n-1) | 0.04 | 0.07 |

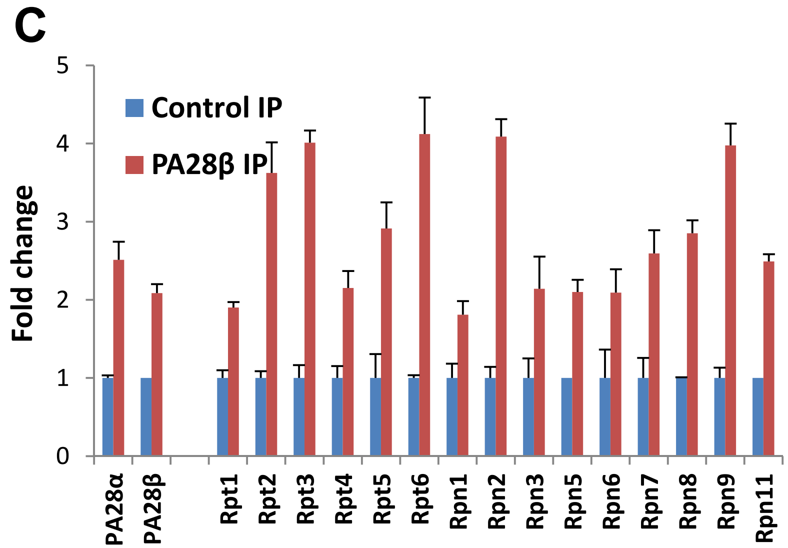


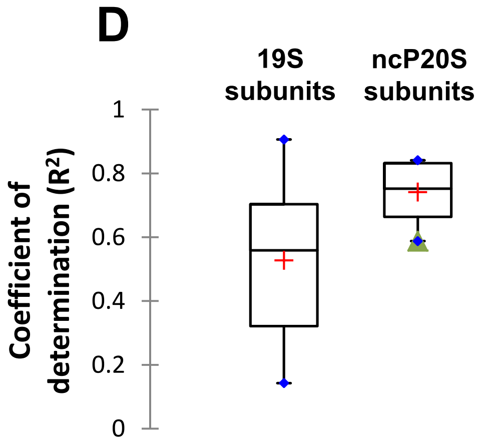


| Statistic for $R^2$ | 19S subunits | 20S subunits |
|---|---|---|
| No. of observations | 324 | 122 |
| Minimum | 0.14 | 0.59 |
| Maximum | 0.91 | 0.84 |
| 1st Quartile | 0.32 | 0.66 |
| Median | 0.56 | 0.75 |
| 3rd Quartile | 0.70 | 0.83 |
| Mean | 0.53 | 0.74 |
| Variance (n-1) | 0.06 | 0.01 |
| Standard deviation (n-1) | 0.24 | 0.10 |

**Figure S1: A- The total pool of 20S proteasome is immunopurified using the MCP21 antibody.** Upper: Chymotrypsin-like (ChT-like) activity measured in the total cell lysate and in the flowthrough after immunopurification (n=3). Lower: Western-blot analyses in the total lysate (Input) and in the flowthrough after proteasome immunopurification (FT) using antibodies directed against GAPDH and 20S proteasome (MCP21 and anti-core alpha subunits). **B- Box-and-whisker plots showing the correlations of abundances, quantified with the $R^2$, between the different subunits of the 19S regulator and of the 20S proteasome in proteasome immuno-precipitates.** The $R^2$ are obtained from the pair wise comparison of protein abundances of core subunits of the 19S regulator (represented by Rpt1-6, Rpn1-3, 5-14) and of the non-catalytic 20S proteasome subunits (ncP20S) (represented by the 11 subunits constitutively found in the 20S CP, α1- α7, β3, β4, β6, and β7). Results were obtained from 324 (19S RP) and 122 (20S CP) observations. Red + and blue dots represent the mean values and the extreme values, respectively. In the table below the graph, detailed statistics for $R^2$ values are indicated. **C- Detection of hybrid proteasome in U937 cells.** Proteins were purified using an antibody directed the PA28β subunit (Cell signaling) and analyzed using quantitative label-free mass spectrometry. The contents of PA28β, PA28α, and 19S RP subunits in the immunopurification with the anti-PA28β antibody were compared with the ones in the control experiment (immunopurification with pre-immunized rabbits antibodies) to obtain a fold change relative to the control. **D- Box-and-whisker plots showing the correlations of abundances, quantified with the $R^2$, between the different subunits of the 19S regulator and of the 20S proteasome non catalytic subunits in the total cell lysates.** The same method as for B- was used.
