# Supplementary material for: Deciphering preferential interactions within supramolecular protein complexes: the proteasome case
Source: Mol Syst Biol. 2015 Jan 5;11(1):771. doi: 10.15252/msb.20145497 (PMC4332148; doi:10.15252/msb.20145497)
Supplement: Supplementary file 2 [file msb0011-0771-sd2.pdf]

Figure S2

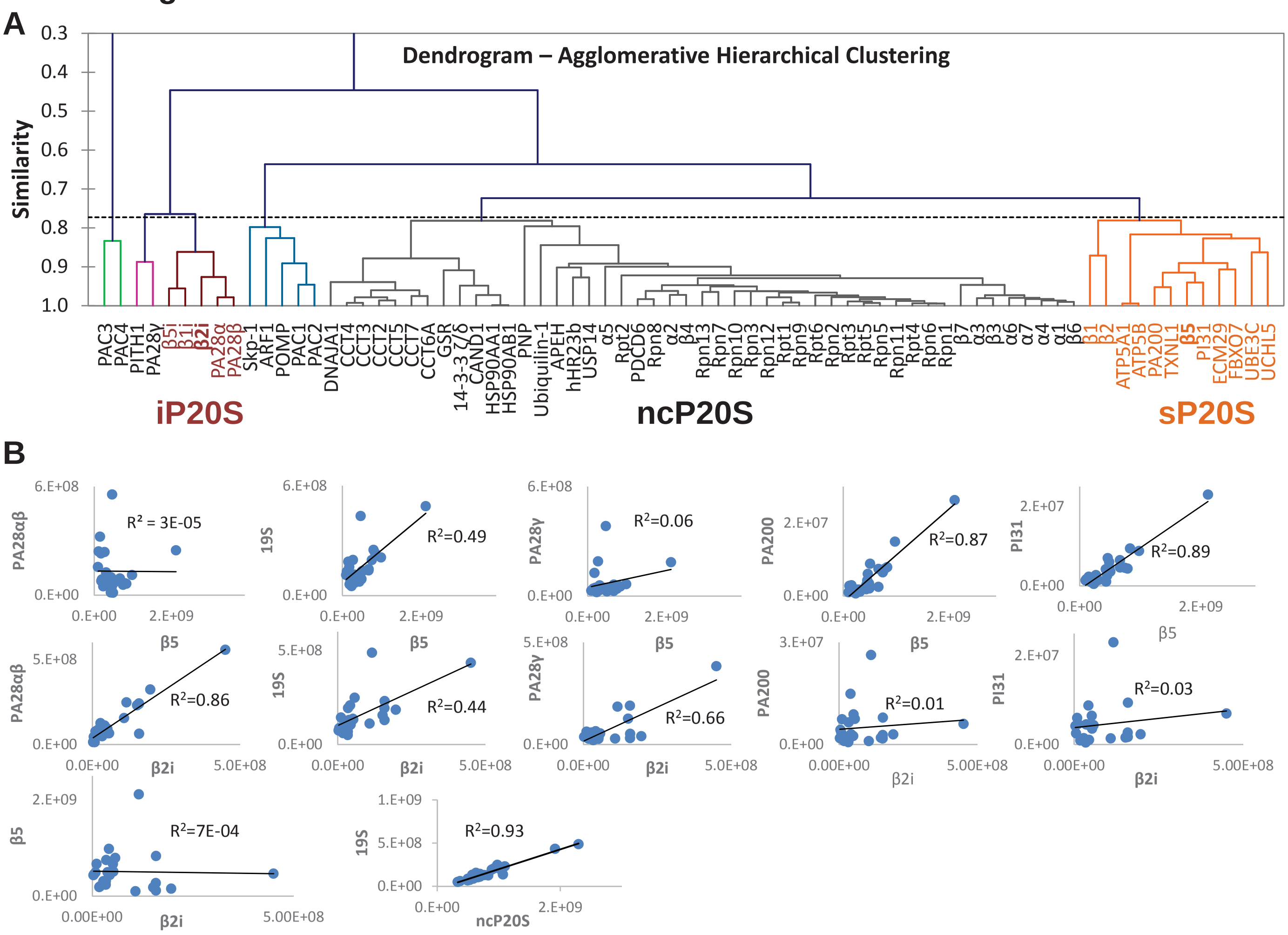

A
Dendrogram – Agglomerative Hierarchical Clustering
Similarity
0.3
0.4
0.5
0.6
0.7
0.8
0.9
1.0
PAC3 PAC4 PITH1 PA28γ β5i β1i β2i PA28α PA28β Skp-1 ARF1 POMP PAC1 PAC2 DNAJA1 CCT4 CCT3 CCT2 CCT5 CCT7 CCT6A GSR 14-3-3 ζ/δ CAND1 HSP90AA1 HSP90AB1 PNP Ubiquilin-1 APEH hHR23b USP14 α5 Rpt2 PDCD6 Rpn8 α2 β4 Rpn13 Rpn7 Rpn10 Rpn3 Rpn12 Rpt1 Rpn9 Rpt6 Rpn2 Rpt3 Rpt5 Rpn5 Rpn11 Rpt4 Rpn6 Rpn1 β7 α3 β3 α6 α7 α4 α1 β6 β1 β2 ATP5A1 ATP5B PA200 TXNL1 β5 PI31 ECM29 FBXO7 UBE3C UCHL5
iP20S
ncP20S
sP20S
B
PA28αβ
β5
R² = 3E-05
19S
R²=0.49
PA28γ
R²=0.06
PA200
R²=0.87
PI31
R²=0.89
PA28αβ
β2i
R²=0.86
19S
R²=0.44
PA28γ
R²=0.66
PA200
R²=0.01
PI31
R²=0.03
β5
β2i
R²=7E-04
19S
ncP20S
R²=0.93

Figure S2: A- The abundances of 73 known PIPs were analyzed using an unsupervised Agglomerative Hierarchical Clustering approach. The clusters nicely grouping the subunits of the iP20S (in dark red), the ncP20S (in black), and the sP20S (in orange) were highlighted. **B- Correlations between the abundances of the iP20S (represented by β2i) or the sP20S (represented by β5) and the 5 main proteasome regulators (PA28αβ, 19S, PA28γ, PA200, and PI31) across the 24 AP-MS experiments.**

PA28αβ profile corresponds to the median of PA28α and PA28β profiles and the 19S profile corresponds to the median of Rpt1-6, Rpn1-3, 5-14 profiles.
