# Supplementary material for: Deciphering preferential interactions within supramolecular protein complexes: the proteasome case
Source: Mol Syst Biol. 2015 Jan 5;11(1):771. doi: 10.15252/msb.20145497 (PMC4332148; doi:10.15252/msb.20145497)
Supplement: Supplementary file 3 [file msb0011-0771-sd3.pdf]

Figure S3

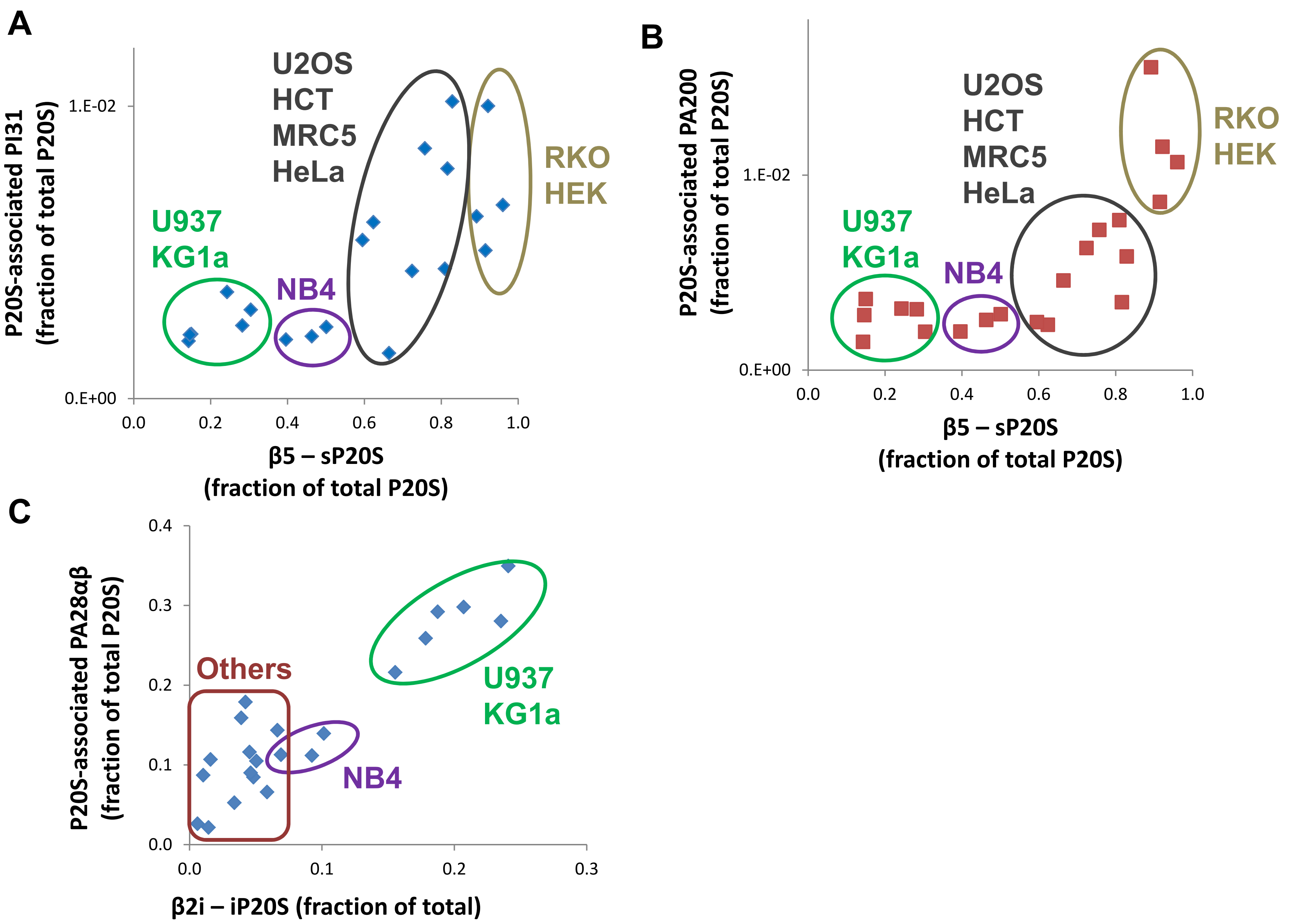
A
U2OS
HCT
MRC5
HeLa
RKO
HEK
U937
KG1a
NB4
P20S-associated PI31 (fraction of total P20S)
1.E-02
0.E+00
0.0
0.2
0.4
0.6
0.8
1.0
β5 – sP20S (fraction of total P20S)
B
U2OS
HCT
MRC5
HeLa
RKO
HEK
U937
KG1a
NB4
P20S-associated PA200 (fraction of total P20S)
1.E-02
0.E+00
0.0
0.2
0.4
0.6
0.8
1.0
β5 – sP20S (fraction of total P20S)
C
P20S-associated PA28αβ (fraction of total P20S)
0.4
0.3
0.2
0.1
0.0
Others
U937
KG1a
NB4
0.0
0.1
0.2
0.3
β2i – iP20S (fraction of total)

Figure S3: Distribution of the PA200, PI31 and PA28αβ regulators associated with the standard or the immunoproteasome in the different cell lines. A, B) The fraction of total P20S (PAI normalized by the median PAI of the 20S proteasome non-catalytic subunits) of the PI31 (A) and PA200 (B) regulators were plotted against the β5 fraction in the different cell lines. C) The fraction of total P20S of the PA28αβ regulator were plotted against the β2i subunit fraction of total P20S in the different cell lines. Data corresponding to the different cell lines were represented on the different graphs.
