# Supplementary material for: Deciphering preferential interactions within supramolecular protein complexes: the proteasome case
Source: Mol Syst Biol. 2015 Jan 5;11(1):771. doi: 10.15252/msb.20145497 (PMC4332148; doi:10.15252/msb.20145497)
Supplement: Supplementary file 4 [file msb0011-0771-sd4.pdf]

Figure S4

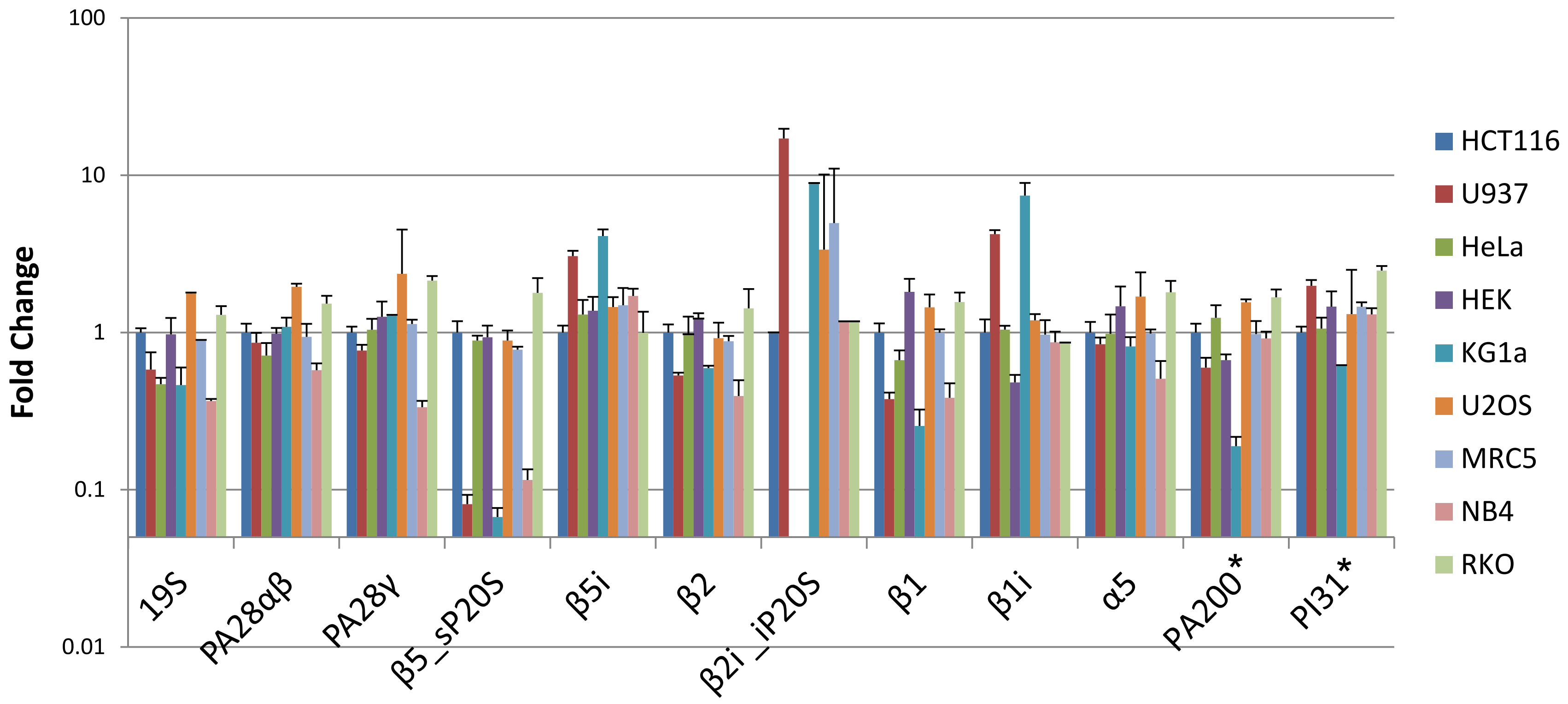

Fold Change
100
10
1
0.1
0.01
19S
PA28αβ
PA28γ
β5_sP20S
β5i
β2
β2i _iP20S
β1
β1i
α5
PA200*
PI31*
HCT116
U937
HeLa
HEK
KG1a
U2OS
MRC5
NB4
RKO

Figure S4: Expression levels of the different proteasome subunits and subcomplexes in the lysates of the nine cell lines. Protein abundances were obtained from label free MS quantification and normalized with the median abundance of all the detected histones. The abundances of 19S and PA28αβ are calculated by meaning the abundances of their corresponding subunits (Rpt1-6, Rpn1-3, 5-14 for the 19S and PA28α, PA28β for PA28αβ). Fold changes compared with the HCT116 cell line are shown (n=3). Proteins with an asterisk were quantified by MRM and normalized with histone H2A type 1-B (as detailed in the Supplementary Materials and Methods). MRM transitions could be unambiguously assigned with the help of co-injected isotope-labeled peptides.
