# Supplementary material for: Deciphering preferential interactions within supramolecular protein complexes: the proteasome case
Source: Mol Syst Biol. 2015 Jan 5;11(1):771. doi: 10.15252/msb.20145497 (PMC4332148; doi:10.15252/msb.20145497)
Supplement: Supplementary file 5 [file msb0011-0771-sd5.pdf]

Figure S5

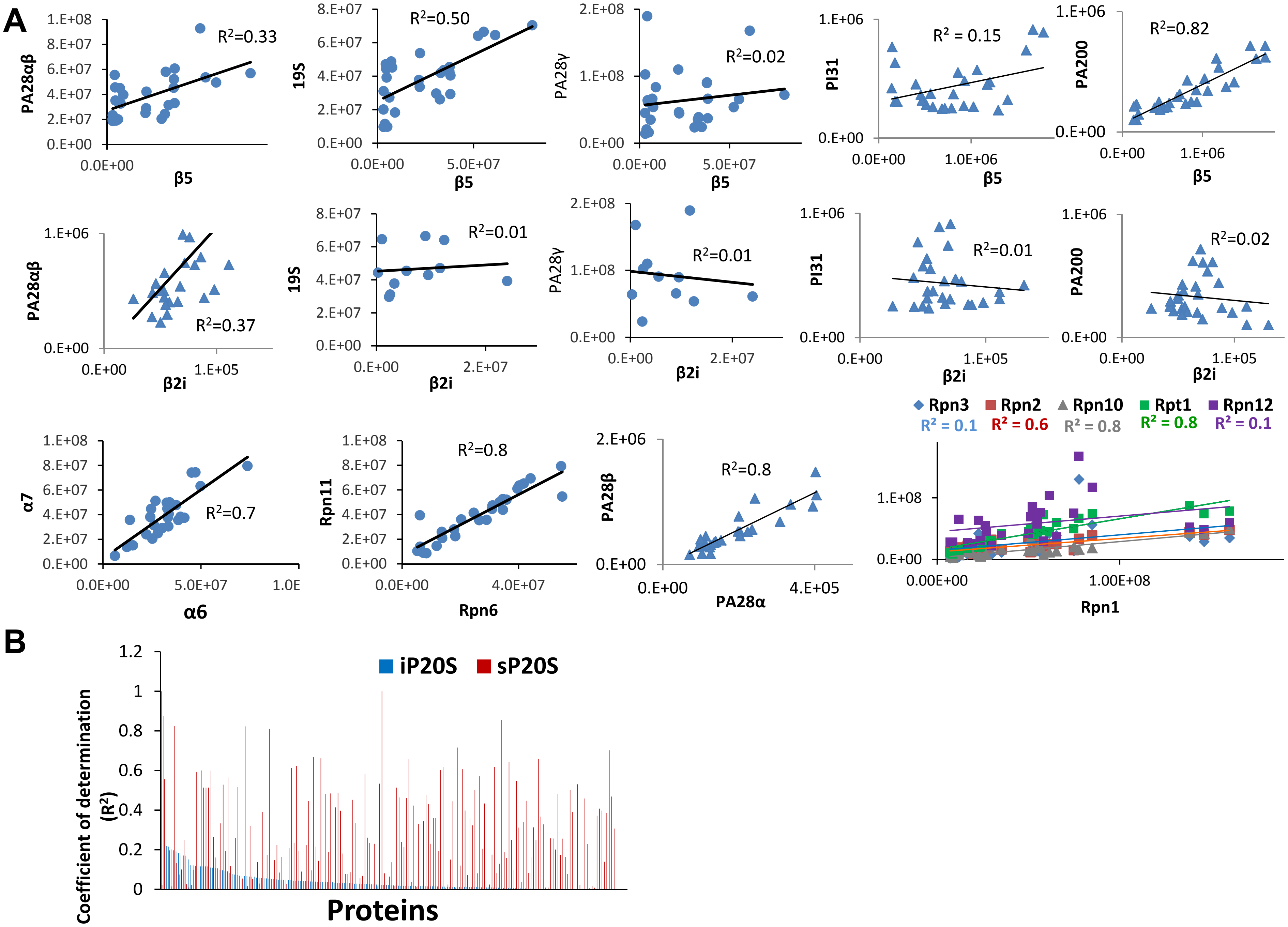

A
PA28αβ
β5
R²=0.33
19S
R²=0.50
PA28γ
R²=0.02
PI31
R² = 0.15
PA200
R²=0.82
β2i
R²=0.37
R²=0.01
R²=0.01
R²=0.01
R²=0.02
α7
α6
R²=0.7
Rpn11
Rpn6
R²=0.8
PA28β
PA28α
R²=0.8
Rpn3 R² = 0.1
Rpn2 R² = 0.6
Rpn10 R² = 0.8
Rpt1 R² = 0.8
Rpn12 R² = 0.1
Rpn1
B
iP20S
sP20S
Coefficient of determination (R²)
Proteins

Figure S5: Correlations of proteins abundances in the total cell lysates of the 9 cell lines

A) Correlations of proteins abundances in the total cell lysates of the 9 cell lines. The abundances of the 5 main proteasome regulators (PA28,βα 19S, PA28 ,γPI31, and PA200) were plotted as a function of the abundances of the iP20S (represented by β2i) or the sP20S (represented by β5). Correlations of abundances of α7 and α6, Rpn6 and Rpn11, PA28β and PA28α, and Rpn1 with Rpt1, Rpn2, Rpn3, Rpn10, and Rpn12**.** PA28αβ abundance corresponds to the median of PA28α and PA28β abundances and the 19S abundance corresponds to the median of Rpt1-6, Rpn1-3, 5-14 abundances. Protein abundances were obtained by calculating the PAI from label free MS quantification (blue circles) or from MRM quantification (blue triangles), as detailed in the Supplementary Materials and Methods.

B) Plot of the R^2 values in the 24 cell total lysates between the iP20S or the sP20S and 193 proteins correlating ($R^2 > 0.8$) with the iP20S, the sP20S or the ncP20S in proteasome complexes.
