# Supplementary material for: Deciphering preferential interactions within supramolecular protein complexes: the proteasome case
Source: Mol Syst Biol. 2015 Jan 5;11(1):771. doi: 10.15252/msb.20145497 (PMC4332148; doi:10.15252/msb.20145497)
Supplement: Supplementary file 6 [file msb0011-0771-sd6.pdf]

Figure S6

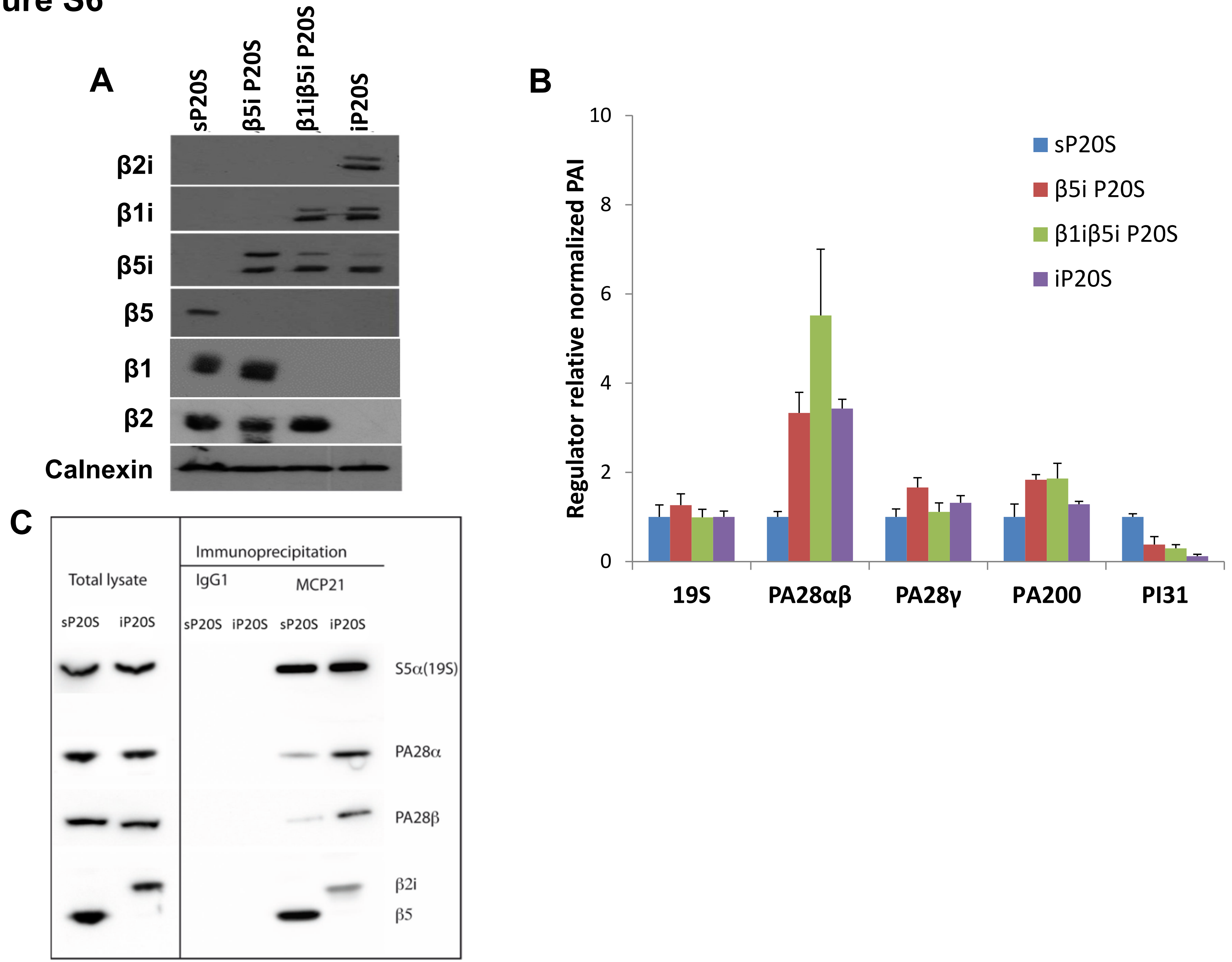
A
sP20S
β5i P20S
β1iβ5i P20S
iP20S
β2i
β1i
β5i
β5
β1
β2
Calnexin
B
Regulator relative normalized PAI
0
2
4
6
8
10
sP20S
β5i P20S
β1iβ5i P20S
iP20S
19S
PA28αβ
PA28γ
PA200
PI31
C
Total lysate
Immunoprecipitation
IgG1
MCP21
sP20S
iP20S
sP20S
iP20S
sP20S
iP20S
S5α(19S)
PA28α
PA28β
β2i
β5

Figure S6: Interaction of the four different 20S proteasome subtypes (sP20S, β5i P20S, β1iβ5i P20S or iP20S) with the main 20S proteasome regulators in HEK EBNA cell lines transfected with β5i (β5i intermediate P20S), β1i and β5i (β1iβ5i intermediate P20S) or β1i, β2i, β5i (iP20S).

A) Immunodetection of the catalytic subunits in the total cell lysates from the four HEK EBNA cell lines expressing only standard catalytic subunits (sP20S), a mixture of standard and catalytic subunits (β5i intermediate P20S and β1iβ5i intermediate P20S), or only immuno catalytic subunits (iP20S). Calnexin is used as a loading control.

B) Relative normalized abundance indexes of proteasome regulators in HEK EBNA cells containing only sP20S, iP20S, β5i intermediate P20S or β1iβ5i intermediate P20S. The normalized abundance indexes for each regulator was set to 1 for standard proteasome conditions (n=4).

C) Comparison of the quantity of 19S and PA28αβ regulators associated with the 20S proteasome in HEK T-Rex cells containing only iP20S or sP20S. Western blot analyses were performed on total cell lysates or proteasome MCP21 immunoprecipitates with antibodies against the S5a (Rpt1 – 19S subunit), PA28α, PA28β, β2i and β5 subunits.
