# Supplementary material for: Deciphering preferential interactions within supramolecular protein complexes: the proteasome case
Source: Mol Syst Biol. 2015 Jan 5;11(1):771. doi: 10.15252/msb.20145497 (PMC4332148; doi:10.15252/msb.20145497)
Supplement: Supplementary file 7 [file msb0011-0771-sd7.pdf]

Figure S7

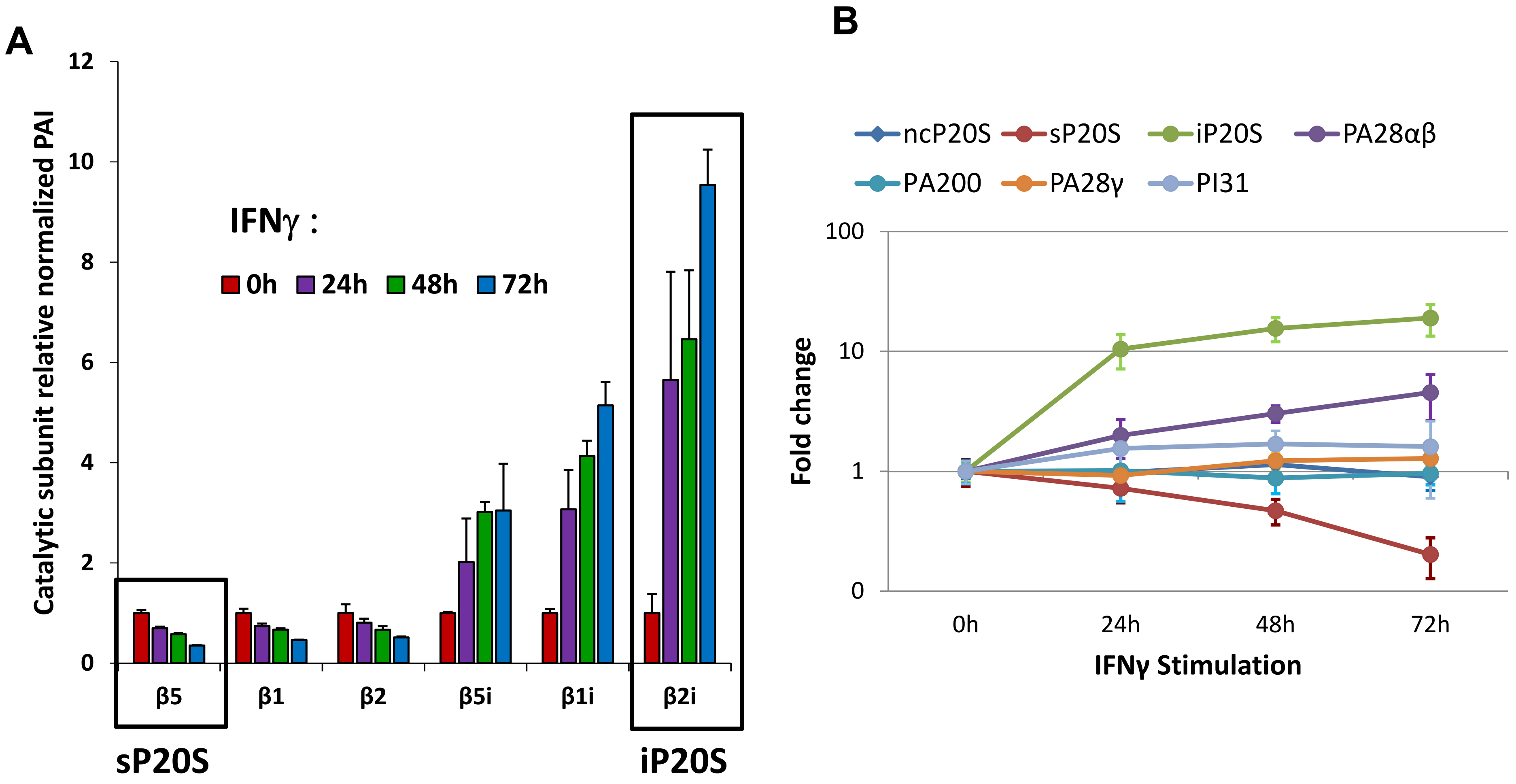

A
Catalytic subunit relative normalized PAI
12
10
8
6
4
2
0
IFNγ :
0h
24h
48h
72h
β5
β1
β2
β5i
β1i
β2i
sP20S
iP20S
B
ncP20S
sP20S
iP20S
PA28αβ
PA200
PA28γ
PI31
100
10
1
0
Fold change
0h
24h
48h
72h
IFNγ Stimulation

Figure S7: sP20S and iP20S associate with a different subset of regulators

A) 20S proteasome subunits dynamics upon IFN-γ kinetic in proteasome immunoprecipitates was measured by label free quantitative proteomic. The normalized abundance index of each catalytic 20S subunit at each time point was compared to the one obtained at the 0h time point to obtain a catalytic subunit relative normalized PAI. The kinetics results for β5 and β2i, representing the sP20S and the iP20S, respectively, are black framed (n=3 per condition).

B) Variations of the expression level of the different proteasome sub-complexes in the total cell lysate and during the IFN-γ kinetic (n=3 per condition). The abundances of ncP20S subunits (median abundance of α2, α7, α4 and β6), sP20S (abundance of β5), iP20S (abundance of β2i), PA28αβ (median abundance of PA28α and PA28β), PA200, PA28γ, and PI31 were obtained by SRM and normalized with the median abundance of histones H1.2 and H2A type 1-B. The abundances of the 19S subunits (Rpt1-6, Rpn1-3, 5-14) were obtained from label free MS quantification and normalized with the median abundance of all the detected histones.
