# Supplementary material for: Deciphering preferential interactions within supramolecular protein complexes: the proteasome case
Source: Mol Syst Biol. 2015 Jan 5;11(1):771. doi: 10.15252/msb.20145497 (PMC4332148; doi:10.15252/msb.20145497)
Supplement: Supplementary file 8 [file msb0011-0771-sd8.pdf]

Figure S8

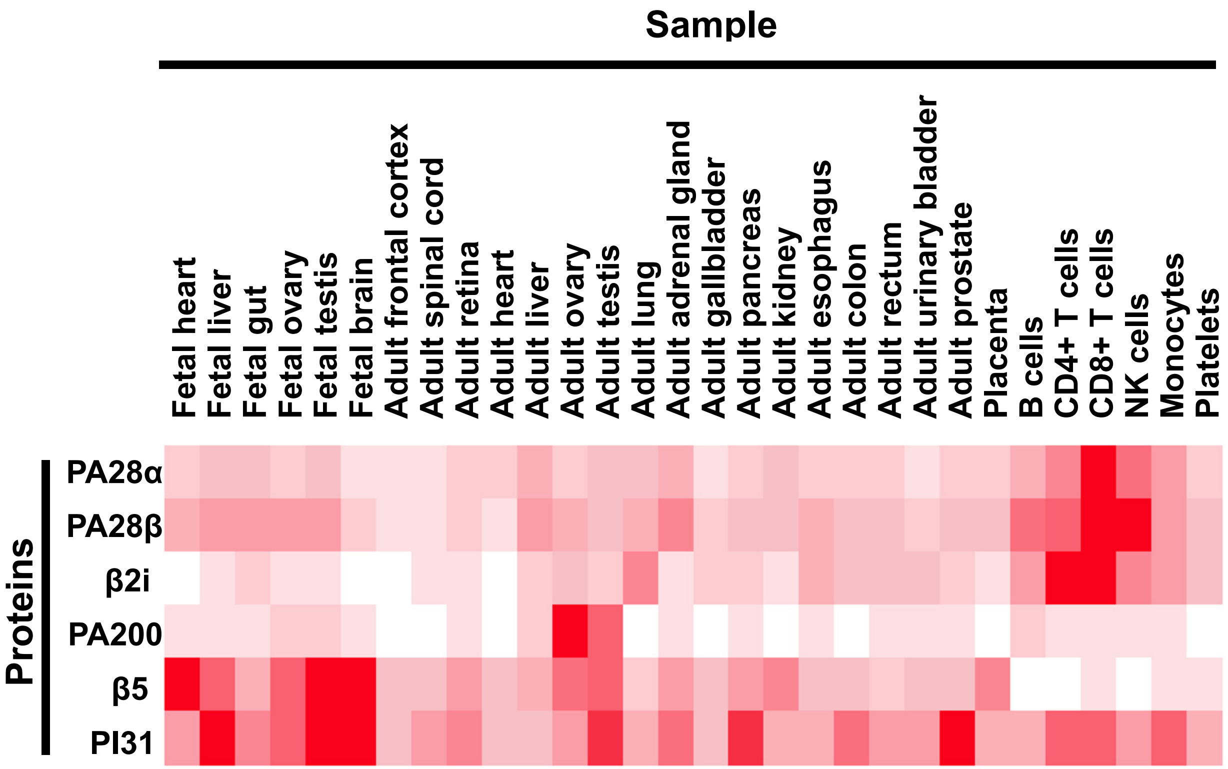
Sample
Fetal heart
Fetal liver
Fetal gut
Fetal ovary
Fetal testis
Fetal brain
Adult frontal cortex
Adult spinal cord
Adult retina
Adult heart
Adult liver
Adult ovary
Adult testis
Adult lung
Adult adrenal gland
Adult gallbladder
Adult pancreas
Adult kidney
Adult esophagus
Adult colon
Adult rectum
Adult urinary bladder
Adult prostate
Placenta
B cells
CD4+ T cells
CD8+ T cells
NK cells
Monocytes
Platelets
Proteins
PA28α
PA28β
β2i
PA200
β5
PI31

Figure S8: Abundances of PA28α, PA28β, β2i, PA200, β5 and PI31 across a panel of 30 histologically normal samples from diverse tissues.

Results were obtained using the Human Proteome Map resource made freely available by Kim et al. (Kim, Pinto et al. 2014). Genes corresponding to PA28α, PA28β, β2i, PA200, β5, and PI31 proteins are PSME1, PSME2, PSMB10, PSME4, PSMB5, and PSMF1, respectively.
