# Supplementary material for: Deciphering preferential interactions within supramolecular protein complexes: the proteasome case
Source: Mol Syst Biol. 2015 Jan 5;11(1):771. doi: 10.15252/msb.20145497 (PMC4332148; doi:10.15252/msb.20145497)
Supplement: Supplementary file 17 [file msb0011-0771-sd17.docx]

**Legends of Supplementary Tables:**

**Supplementary Table S1 :** List and detailed quantification report of the proteins quantified for the PCP- MS analysis of glycerol gradient separated protein complexes presented in Figure 1 & 2.

**Supplementary Table S2 :** List and detailed quantification report of the known 73 PIPs used in Figures 4A&B and Figure S2A.

**Supplementary Table S3 :** Quantification report of protein expression in the 9 cell lines obtained by label-free quantification and by SRM. Data used for Figures S4 & S5A.

**Supplementary Table S4 :** List and detailed quantification report of the 193 putative PIPs used in Figure 4C.

**Supplementary Table S5 :** List and detailed quantification report of the proteins quantified in the proteasome immunoprecipitates from HEK EBNA cells expressing either standard proteasome or immunoproteasome. Data used for Figure 5B and Figure S6B.

**Supplementary Table S6 :** List and detailed quantification report of the proteins quantified in the proteasome immunoprecipitates from IFNγ-treated HeLa cells. Data used for Figure 5D and Figure S7A.

**Supplementary Table S7 :** Quantification report of protein expression in the HeLa cells during the IFNγ stimulation and obtained by SRM. Data used for Figures S7B.

**Supplementary Table S8 :** Detailed description of the files (raw files and Mascot result files) and the associated samples names deposited in ProteomeXchange (dataset identifier PXD001043).
