# Supplementary material for: Deciphering preferential interactions within supramolecular protein complexes: the proteasome case
Source: Mol Syst Biol. 2015 Jan 5;11(1):771. doi: 10.15252/msb.20145497 (PMC4332148; doi:10.15252/msb.20145497)
Supplement: Supplementary file 18 [file msb0011-0771-sd18.pdf]

Supplementary Materials and Methods

Targeted mass spectrometry analysis – Multiple reaction Monitoring (MRM) for measurement of expression levels of proteins. Proteins samples were prepared as described in the main Material and Methods section. Each dried peptides sample was suspended in 14µL of a solution containing 2% acetonitrile, 0.05% TFA, and synthetic stable isotope-labeled peptides (partially purified PEPotec synthetic containing on their C-terminus an ^{15}N and ^{13}C-labeled arginine or lysine residue, ~250-500 fmol/µL, Thermo Scientific) corresponding to the light proteotypic peptide sequences analyzed. Five µL (about 2µg of equivalent proteins) was then loaded on the system and analyzed on a hybrid triple quadrupole-ion trap mass spectrometer 5500 QTrap (AB Sciex) equipped with a nanoelectrospray ion source coupled to an Ultimate3000 system (Dionex) for chromatographic peptide separation using a 60 min gradient from 0 to 50% of solvent B (80% acetonitrile, 0.2% formic acid) at a flow rate of 300 nL/min. Spray voltage was set at 2800V, curtain gas at 20 psi, nebulizer gas at 6 psi, interface heater temperature at 150°C, and cycle time at 3 s. Peptides were loaded onto a C18 precolumn (300 µm ID x 5 mm, Thermo Scientific) at 20 µL/min in 2% acetonitrile, 0.05% trifluoroacetic acid. After 5 min of desalting, the precolumn was switched online with the analytical C-18 column (75 µm ID x 15 cm, Acclaim® PepMap RSLC nanoViper 2µm, 100Å, Thermo Scientific) and equilibrated in solvent A (5% acetonitrile, 0.2% formic acid). Collision energies (CEs) have been optimized to reach the maximal sensitivity.

SRM transitions are as follow: α2 (SILYDER: 448.23/695.33, 448.23/582.25, 448.23/304.16); α7 (AVENSSTAIGIR: 609.33/345.22, 609.33/804.46, 609.33/918.50); α4 (ALLEVVQSGK: 550.82/575.31, 550.82/803.43, 550.82/916.51); β6 (GAVYSFDPVGSYQR: 773.37/806.41, 773.37/921.44, 773.37/1155.54); β5 (AIYQATYR: 493.26/401.20, 493.26/510.27, 493.26/801.39, DAYSGGAVNLYHVR: 507.92/543.30, 507.92/586.81, 507.92/668.34, VSSDNVADLHEK: 438.55/564.27, 438.55/607.79, 438.55/712.36); β2i (IHFIAPK: 413.25/575.35, 413.25/356.71, 413.25/251.15, LPFTALGSGQDAALAVLEDR: 1022.54/957.54, 1022.54/702.38, 1022.54/532.27, 682.03/707.37,

682.03/756.91, 682.03/813.45); PA200 (SLNLPVGSSQVLVPR: 783.45/1138.66, 783.45/371.24, 783.45/272.17, ALPGVDPNDFSK: 630.32/822.36, 630.32/707.33, 630.32/538.26, NDLTEVER: 488.24/746.40, 488.24/633.32, 488.24/532.27, LFDDLAEK: 475.74/837.40, 475.74/690.33, 475.74/575.30); PA28γ (SNQQLVDIIEK: 643.85/829.0, 643.85/716.42, 643.85/617.35, TVESEAASYLDQISR: 834.91/1052.54, 834.91/981.50, 834.91/894.47, 556.94/731.40, 556.94/618.32, 556.94/375.23, LIISELR: 422.27/617.36, 422.27/504.28, 422.27/227.17); PI31 (N-acetAGLEVLFASAAPAITC[CAM]R: 894.97/859.44, 894.97/788.41, 894.97/717.37, IVSGIITPIHEQWEK: 875.48/1167.58, 875.48/1066.53, 875.48/769.40, 583.99/1167.58, 583.99/1066.53, 583.99/769.40); PA28α (TENLLGSYFPK: 634.83/811.43, 634.83/698.35, 634.83/571.31, ISELDAFLK: 518.29/922.49, 518.29/835.46, 518.29/706.41, YFSER: 351.17/538.26, 351.17/391.19, 351.17/311.14); PA28β (QVEVFR: 389.22/550.30, 389.22/421.25, 389.22/322.19, QNLFQEAEEFLYR 843.91/1184.56, 843.91/1056.50, 843.91/927.46, VEAFQTTISK: 562.30/895.49, 562.30/824.45, 562.30/229.12); Histone H1.2 (ASGPPVSELITK 599.84/886.52, 599.84/564.32, 599.84/520.80, 599.84/492.29, SGVSLAALK 423.26/701.46, 423.26/602.39, 423.26/351.23, 423.26/244.13); Histone H2A type 1-B (AGLQFPVGR: 472.77/575.33, 472.77/428.26, 472.77/408.74, 472.77/517.28, HLQLAIR: 425.77/713.47, 425.77/600.38, 425.77/472.32, 425.77/251.15). Transitions could be unambiguously assigned with the help of co-injected isotope-labeled peptides.
