# Supplementary material for: Deciphering preferential interactions within supramolecular protein complexes: the proteasome case
Source: Mol Syst Biol. 2015 Jan 5;11(1):771. doi: 10.15252/msb.20145497 (PMC4332148; doi:10.15252/msb.20145497)
Supplement: Supplementary file 19 [file msb0011-0771-sd19.pdf]

Deciphering preferential interactions within supramolecular protein complexes: the proteasome case

Bertrand Fabre, Thomas Lambour, Luc Garrigues, François Amalric, Nathalie Vigneron, Thomas Menneteau, Alexandre Stella, Bernard Monsarrat, Benoît Van den Eynde, Odile Burlet-Schiltz, Marie-Pierre Bousquet-Dubouch

Corresponding author: Marie-Pierre Bousquet-Dubouch, CNRS - IPBS

Review timeline:

Submission date:	12 June 2014
Editorial Decision:	24 July 2014
Revision received:	22 October 2014
Editorial Decision:	17 November 2014
Revision received:	21 November 2014
Accepted:	03 December 2014

Editor: Maria Polychronidou

Transaction Report:

(Note: With the exception of the correction of typographical or spelling errors that could be a source of ambiguity, letters and reports are not edited. The original formatting of letters and referee reports may not be reflected in this compilation.)

1st Editorial Decision 24 July 2014

Thank you again for submitting your work to Molecular Systems Biology. We have now heard back from two of the three referees who agreed to evaluate your manuscript. Since their recommendations are fairly similar, I prefer to make a decision now rather than further delaying the process. As you will see from the reports below, the referees acknowledge that the presented findings are potentially interesting. However, they raise a series of concerns, which should be carefully addressed in a revision of the manuscript.

Without repeating all the comments listed below, one of the more fundamental issues refers to the need to include further experimentation and additional analyses in order to robustly support the main conclusions. Reviewer #1 offers constructive suggestions in this regard.

On a more editorial level, we would like to draw your attention to the following:
- In line with the comment of reviewer #1, we would ask you to streamline the Discussion.

Reviewer #1:

The manuscript by Fabre et al. describes a newly developed method combining affinity purification and protein correlation profiling associated with high resolution mass spectrometry. Using this proteomics approach the authors investigated the composition of proteasome complexes in different cell lines or under different conditions. Subsequently, they correlated the preferential association of

© European Molecular Biology Organization

the two major 20S core subcomplexes (standard and immuno-20S core proteasome) with distinct regulator complexes. In their correlation calculations the authors found that sp20S cores preferentially interact with PA200 or PI31, whereas ip20S was predominantly associated with the 11S regulator.

Although the topic is very interesting and relevant the authors failed to fully support their key conclusions convincingly with the data shown. The experiments are carefully designed and performed, however the analysis of the data suffers from miss-interpretation of data by exclusion of the biological context and previous knowledge. The manuscript would benefit from altered interpretation of the data within the biological setting of their cell models and in the context of published literature. Some experiments should be also supported by alternative biochemical data sets (see detailed concerns). Such improved manuscript will then add to the conceptual advance in the understanding of proteasome heterogeneity and their function in protein homeostasis under different conditions or in certain tissues. The broad audience of the MSB journal will also appreciate the focus on the natural context of heterogeneous proteasome populations in different cell types.

Major points:
The authors should draw their attention to the origin of their cell lines with respect to cell types naturally express high amounts of immunoproteasomes and PA28ab such as cells of lymphoid or myeloid origin.
As a proof of principle for preferred interaction of ip20S with PA28 the authors should recalculate their data only for the NB4 cell line in comparison to HEK293 cells. This lymphoma cell line should mainly contain immunoproteasomes. Using the described calculation the correlation between beta2i and PA28ab should be higher compared to other cells with lower ip20S content and would strengthen their key conclusions. Vice versa this recalculation should also work for the preferential interaction of sp20S with PI31 or PA200 in the context of HEK293 cells.
Generally expression controls (immunoblot) for representative subunits of the standard- and the immunoproteasome as well as the 11S or 19S regulators are required to convince this reviewer that the differential interactions are not caused by higher or lower expression of the complexes.

Proteasome assembly chaperones (Pac1-4, POMP, and probably also others) are exclusively found in proteasome assembly intermediates (see the work of Mark Hochstrasser, Jurgen Dohmen, Anne Peyroche, Shigeo Murata, Elke Kruger, Peter Kloetzel and others). Assembly intermediates are proteasome precursor complexes and inactive per definition. Such inactive intermediates cannot contribute to ubiquitin dependent protein break-down in a cell. Thus, the authors should recalculate their data without these precursor complexes, which can be easily excluded by their size and lower density (smaller than 20S) using their gradient fractionation. Otherwise, their clustering into the selected groups is incorrect. Nevertheless, the presence of assembly chaperones may indicate the frequency of proteasome formation of a certain proteasome type. What does the term ncP20S in this context mean? An active 20S core complex also contains alpha subunits, which are not catalytically active on their own but required as scaffolds in the complex.

The authors ignore the existence of so-called hybrid proteasomes, in which the 20S core complex (most likely immunoproteasome 20S) is associated with the 19S regulator on one side and with the 11S regulator (PA28 a, b) on the other (Tanahashi N et al., JBC 2000). Can the authors conclude from their correlations whether hybrid proteasomes exist as one entity in their preparations? Here, a supportive biochemical control experiment (native PAGE analyses and immunoblotting stained for PA28 and a 19S subunit or immunoprecipitation and counterstaining for both) should be performed to clarify this.
This reviewer also wonders why the authors do not refer to their own work on intermediate type proteasomes (containing standard as well as immuno-active sites)? Is the method used suitable to detect this subtype? These proteasomes also contribute to the heterogeneity and should be mentioned and at least discussed in this context.

The experiments in Figure 5 are nice to support the drawn conclusions from the first more systems biology approach. However, to draw these conclusions an expression control (immunoblot) for representative regulator subunits is required (PA28, 19S).Why the authors did not use beta5i as the immunoproteasome counterpart of beta5?

Minor points
The discussion is too long (6 pages for 5 Figures).
Sometimes the references are a bit strange and not up to date. As an example there are very nice structural studies of human 26S proteasomes by the Morris group (da Fonseca et al., Mol Cell 2012) showing the gate opening and the specific interaction sites.
A better explanation for the content of the supplemental tables would be helpful for readers.

Reviewer #2:

This manuscript describes a combination of AP-MS and PCP to characterize the normal and immunoproteasomes from many different cell lines to look at the differences in stoichiometry of attached regulatory molecules. It is nicely done presents a compelling story, although there is not really any mechanisms being tests. That may not be essential though, in this case.

Concerns
- I am concerned that the high correlation described for Figure 3 may be an artefact of the immunopurification approach used. The details of the IP conditions do not seem to be described in the Methods for one thing but if the same antibody is used for each cell line (as I presume it was) then perhaps the antibody can only pull down certain configurations of the proteasome that would have similar abundances of their subunits? This could be addressed with alternative antibodies or by comparison to a different method, such as PCP

- Mechanisms: I am not a proteasome expert but it would seem that an extension of this work could be in vitro assays of proteasome function are tested with the various combinations of regulatory proteins identified here. I appreciate though that such assays are not yet available, at least where additional individual components can be added or subtracted.

- I'm not sure that the field needs another acronym, especially one as hard to remember as "PAC-AP-HR-MS". Since this is not a methods paper, I suggest aspect of it just be removed since it doesn't really add anything. In addition, focusing on the High Resolution aspect of the MS seems a little passÈ since virtually all proteomics is done with high res instruments now (Orbitraps, QTOFs). Gone (thankfully) are the days when 3D or 2D traps dominated!

Additional correspondence 14 August 2014

We have now received a very late report, from the third referee who was asked to evaluate your study.

The comments of this referee are pasted below for you reference. We would like to ask you to also address this referee's concerns in the revision of your work and to include the related information in the point-by-point response.

Thank you once again for submitting your work to MSB and we are looking forward to receiving your revised work.

Reviewer #3

Fabre et al investigate proteins associated with the proteasome and immunoproteasome by mass spectrometric techniques. Specifically, they combine correlation profiling, where successive fractions are analyzed by uantitative MS to obtain characteristic profiles, with pulldowns, a technique which they call "Protein Abundance Correlation associated to Affinity Purification and High Resolution Mass Spectrometry" (note that this would be called 'associated with' to be grammatically correct).

Overall, the ideas and the results are quite interesting. The major shortcoming is that this technique

is demonstrated on the proteasome, which is extremely abundant and one of the easiest complexes to work on. It is not clear that there would be many other complexes where this technique would work. Furthermore, while the combination of approaches is novel, none of the individual parts are. Therefore a big part of the novelty would have to be in the new associations of factors to the proteasome and immunoproteasome. Of course, it would have been nice if these novel associations had been tested by independent means, but I realize that this may be difficult.

Specific points:
The paper seems to excessively long and this itself makes the logic difficult to follow. As an example, one doesn't need to summarize the findings at the end of the introduction so extensively.
Use of formaldehyde cross-linking before protein correlation profiling is interesting.
Can the authors compare results with and without doing this, so we know what factors are retained only due to the crosslinking? (Maybe this is described in their Fabre 2013 or 2014 paper, but most readers would not look this up.)
People usually use just the term R2 instead of 'coefficient of determination' The authors mention in the discussion that most PIPs were not identified in this study 'probably because of the low fractionation level used'. This is a pity and seems to contradict what the authors say in the beginning of the results where they have 120 PIPs. An alternative explanation would be that many of the previously reported PIPs were actually artifacts. If the technique introduced here is more comprehensive as the authors claim, could it not shed light on which previously reported PIPs are probably spurious?
If the authors want to use the results of the Kim et al. 2014 study, this should be folded into the results section.
Figures would look nicer in color (i.e. figure 1).
Check grammar and spelling in the manuscript (i.e. Protein Abondance Index in figure 3). Also the PAI paper is not cited. The authors use comma "," where they should use period "." to denote the decimal point.

1st Revision - authors' response 22 October 2014

(see next page)

iPBS
Institut de Pharmacologie et de Biologie Structurale
www.ipbs.fr
205 route de Narbonne
31077 Toulouse Cedex04
T. 33-5 61 17 59 18
F. 33-5 61 17 59 93
UMR 5089

Reviewer #1:

MSB-14-5497

The manuscript by Fabre et al. describes a newly developed method combining affinity purification and protein correlation profiling associated with high resolution mass spectrometry. Using this proteomics approach the authors investigated the composition of proteasome complexes in different cell lines or under different conditions. Subsequently, they correlated the preferential association of the two major 20S core subcomplexes (standard and immuno-20S core proteasome) with distinct regulator complexes. In their correlation calculations the authors found that sp20S cores preferentially interact with PA200 or PI31, whereas ip20S was predominantly associated with the 11S regulator.
Although the topic is very interesting and relevant the authors failed to fully support their key conclusions convincingly with the data shown. The experiments are carefully designed and performed, however the analysis of the data suffers from miss-interpretation of data by exclusion of the biological context and previous knowledge. The manuscript would benefit from altered interpretation of the data within the biological setting of their cell models and in the context of published literature. Some experiments should be also supported by alternative biochemical data sets (see detailed concerns). Such improved manuscript will then add to the conceptual advance in the understanding of proteasome heterogeneity and their function in protein homeostasis under different conditions or in certain tissues. The broad audience of the MSB journal will also appreciate the focus on the natural context of heterogeneous proteasome populations in different cell types.

Major points:
The authors should draw their attention to the origin of their cell lines with respect to cell types naturally express high amounts of immunoproteasomes and PA28ab such as cells of lymphoid or myeloid origin.
As a proof of principle for preferred interaction of ip20S with PA28 the authors should recalculate their data only for the NB4 cell line in comparison to HEK293 cells. This lymphoma cell line should mainly contain immunoproteasomes. Using the described calculation the correlation between beta2i and PA28ab should be higher compared to other cells with lower ip20S content and would strengthen their key conclusions. Vice versa this recalculation should also work for the preferential interaction of sp20S with PI31 or PA200 in the context of HEK293 cells.

The method is based on performing correlations of abundances of proteasome subtypes and regulators using all the data from the 24 immunopurifications (2 to 3 proteasome IPs are performed per cell line and 9 cell lines were studied). Doing this in cell types containing highly varying amounts of 20S proteasome forms is much more statistically powerful than comparing proteasome composition in only 2 cell lines. However, we agree that more attention should be paid to the origin of the cell lines. To answer this interesting point, a new figure E3 was obtained from the data. As shown on new Figure E3-C, the three lymphoid and myeloid cell lines (U937, KG1a and NB4) display high amounts of immunoproteasome (the β2i subunit corresponds to 10 to 30% of the

iPBS
Institut de Pharmacologie et de Biologie Structurale

www.ipbs.fr

205 route de Narbonne
31077 Toulouse Cedex04

T. 33-5 61 17 59 18
F. 33-5 61 17 59 93

UMR 5089

total 20S proteasome) and associated PA28αβ activator, compared to the other cell lines analyzed. These three lymphoid and myeloid cell lines also show the lowest fractions of standard proteasome (the β5 subunit corresponds to 20 to 50% of the total 20S proteasome) and associated PI31 and PA200 regulators, compared to the other cell lines (Figure E3-A and E3-B). This has been added in the result part of the revised manuscript (page 14, line 1) "As far as the origin of the cell lines is considered, the three lymphoid and myeloid cell lines (U937, KG1a, and NB4) display, as expected, the highest amounts of immunoproteasome (the β2i subunit corresponds to 10 to 30% of the total P20S) and P20S-associated PA28αβ activator (Figure E3C), and the lowest fractions of standard proteasome (20 to 50% of the total P20S) (Figure E3A-B) and P20S-associated PA200 and PI31 regulators, compared to the other cell lines.**" and Figure E3 has been introduced. Our correlation data are thus in accordance with previous biological knowledge on cellular-dependant proteasome composition.**

Generally expression controls (immunoblot) for representative subunits of the standard- and the immunoproteasome as well as the 11S or 19S regulators are required to convince this reviewer that the differential interactions are not caused by higher or lower expression of the complexes.

To answer the reviewer's concern, two new experiments were performed:

1- Expression levels of representative proteins of the sP20S, iP20S, and of 11S, 19S, PA200, and PI31 regulators were determined in total cell lysates using quantitative mass spectrometry, as shown in revised Figure E4. Protein complexes abundances were normalized with the abundance of histones (as loading control, similarly as it would be done by immunoblot). We preferred mass spectrometry because it constitutes a multiplexed quantitative approach, allowing the simultaneous analysis of all these proteins in a single experiment, and also because efficient antibodies are not available for all the subunits analyzed.

- **As expected, the three lymphoid and myeloid cell lines (U937, KG1a and NB4) express the lowest quantities of β5, β2, and β1 subunits and the highest levels of β2i, β5i, and β1i, although it is less evident for NB4 cells. This can be explained by the fact that PML/RARα is expressed in NB4 but not in KG1a and U937 cell lines (Grande et al, 2001). A recent study has shown that the PML/RARα fusion protein represses the transcription of the immuno subunits (Yang et al, 2014).**
- **Concerning proteasome regulators, variations in expression levels can be observed in the different cell lines (Figure E4), but these appear not to be related to their levels of association with the 20S proteasome. For instance, the PA28αβ regulator is highly co-immunopurified with the 20S proteasome in U937 and KG1a but not expressed in higher levels in these cell lines compare to the 7 seven other cell lines studied (Figure E4). This result thus confirms preferential interactions of PA28αβ with the immunoproteasome**

iPBS
Institut de Pharmacologie et de Biologie Structurale
www.ipbs.fr
205 route de Narbonne
31077 Toulouse Cedex04
T. 33-5 61 17 59 18
F. 33-5 61 17 59 93
UMR 5089

(which is much more abundant in U937 and KG1a than in the other cell lines, Figure E3C). The same trend is observed in the HEK T-Rex cells expressing either the standard catalytic subunits or the immuno catalytic subunits: equivalent amounts of PA28α and PA28β are indeed observed in the cell lysates but higher proportions of PA28α and PA28β are measured in association with the P20S in the cell lines containing iP20S, Figure E6C). This results is in accordance with our observation that a part of the cellular subunits composing these regulators are not associated with 20S proteasome, as shown in the figures below where the total cell lysate of formaldehyde-crosslinked cells (U937, HeLa, and HCT116) and the flow-through after MCP21-immunopurification of proteasomes (FT) were quantified either by immunodetection of SDS-PAGE separated proteins or by label-free MS quantification (equal volumes of "Input" and "FT" samples were analyzed):

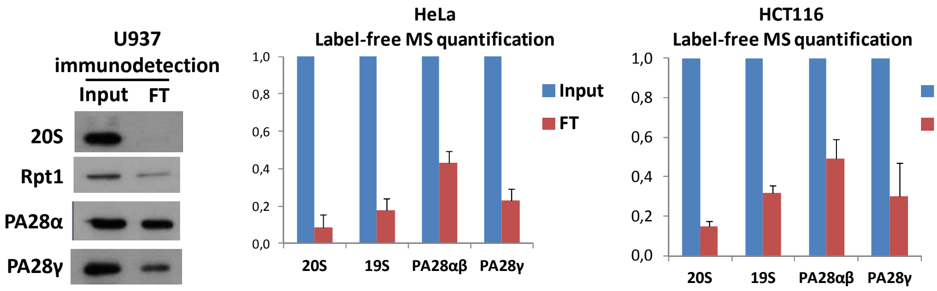

2- To answer more accurately to the reviewer concern, we quantified the correlations of abundances between proteasome subunits and proteasome regulators in the total cell lysates by quantitative mass spectrometry (Figure E5A), as already performed with the abundances measured in proteasome immunoprecipitates (Figure E2B). As shown in the added figure E5A, the expressions of some subunits belonging to the same complex were found to correlate quite well ($R^2 = 0.7$ for the correlation between α6 and α7 expression levels, $R^2 = 0.8$ between Rpn6 and Rpn11 expression levels and $R^2 = 0.8$ between PA28α and PA28β expression levels). However, many other subunits expression levels could not be correlated, such as the ones among 19S subunits (Figure E1D & E5A) or, more importantly, the ones between β2i and PA28αβ ($R^2 = 0.37$) and between β5 and PI31 ($R^2 = 0.15$) (Figure E5A). This is not the case when plotting the abundances of these proteins in the proteasome immunoprecipitates (Figure E2B).

<u>To conclude, these results thus show that the preferential interactions described in this study are not caused by correlation of the proteins expression levels but rather by incorporations of subunits within a common specific proteasome complex form. Only the expression levels of PA200 and β5 are correlated ($R^2 = 0.82$; Fig. E4-A), possibly explaining the common clustering of these proteins in purified proteasomes</u>. These information have been incorporated in the revised manuscript (Page 14, line 6): "Besides, expression controls show that the differential interactions observed within

proteasomes seem not be caused by higher or lower expressions of the subunits forming these complexes (Figure E4 – Expanded Table 3). To check more accurately this point, the expression levels of proteasome and RPs subunits were plotted (Figure E5A), as previously performed for subunits abundances measured in proteasome immunoprecipitates (Figure E2B – Expanded table 3). Noteworthy, some of the correlations measured in purified proteasomes were conserved in total cell lysates (for instance, the good correlation between the 20S proteasome α6 and α7 subunits, R^2 = 0.8, or the one between PA28α and PA28β, R^2 = 0.8). However, many other subunits expression levels could not be correlated, such as the ones among 19S subunits (Figure E1D & E5A) or, more importantly, the ones between β2i and PA28αβ (R^2 = 0.37) and between β5 and PI31 (R^2 = 0.15) (Figure E5A). PA200's expression level is however correlated with the one of β5 (R^2 = 0.82)." **and three new expanded figures have also been added (Figure E3, Figure E4, and Figure E5A).**

iPBS
Institut de Pharmacologie et de Biologie Structurale
www.ipbs.fr
205 route de Narbonne
31077 Toulouse Cedex04
T. 33-5 61 17 59 18
F. 33-5 61 17 59 93
UMR 5089

Another interesting finding highlighted by these correlations of abundances is that some subunits of the 19S regulator have very different expression levels compared to other subunits of the same complex (for instance, see the correlations between Rpn1 and Rpn3 (R^2=0.1), Rpn2 (R^2=0.6), Rpn10 (R^2=0.8), Rpt1 (R^2=0.8), or Rpn12 (R^2=0.1) on Figure E5-A), showing that the expression levels of subunits belonging to the same complex are not necessarily correlated (the distribution of the R^2 across the abundances of the 19S subunits in the cell lysates is very dispersed, as presented on Fig. E1-C). Thus, the purification of the complex is necessary to highlight correlations between subunits in a particular complex. This result has been added in the revised manuscript (page 10, line 7): "Moreover, the cellular expression levels of many 19S subunits and of some 20S subunits are not correlated (Figure E1D), contrary to the abundances of these proteins in immuno-purified proteasomes (Figure E1B), showing that the immuno-enrichment step is required to highlight subunits interactions within a protein complex."

Proteasome assembly chaperones (Pac1-4, POMP, and probably also others) are exclusively found in proteasome assembly intermediates (see the work of Mark Hochstrasser, Jurgen Dohmen, Anne Peyroche, Shigeo Murata, Elke Kruger, Peter Kloetzel and others). Assembly intermediates are proteasome precursor complexes and inactive per definition. Such inactive intermediates cannot contribute to ubiquitin dependent protein break-down in a cell. Thus, the authors should recalculate their data without these precursor complexes, which can be easily excluded by their size and lower density (smaller than 20S) using their gradient fractionation.

- **Proteasome assembly intermediates indeed exhibit lower sedimentation coefficients than mature 20S proteasome and are associated to proteasome assembly chaperones. In the experiment presented in Figures 1 & 2, low MW proteins were first eliminated by ultrafiltration (cut-off of 100kDa) and protein complexes remaining in the UF retentate were then separated according to their sedimentation coefficient using glycerol gradient ultracentrifugation. This experiment therefore enables to**

separate proteasome assembly intermediates from active and mature proteasomes. No 20S proteasome chaperone is reliably detected by MS in this experiment, which strengthens this conclusion. Even in the absence of these precursor complexes, a nice correlation is observed between the β2i subunit (specific of the immuno 20S proteasome) and the PA28αβ regulator, as presented in Figure 2.

- **As far as the data obtained from proteasome immunopurifications are concerned (Figures 3-5), the identification of proteasome chaperones indeed shows that proteasome assembly intermediates were purified. The abundances of 20S subunits indeed include the fractions of subunits involved in proteasome assembly intermediates. However, these 20S proteasome assembly intermediates represent a very low fraction of the total 20S proteasome pool, around 6% in average in the 9 cell lines (Fabre et al, 2014). Thus, they should not affect the quality of the data. If so, the 20S proteasome non catalytic subunits (ncP20S) could not have been so well correlated with the subunits of the 19S regulator (Figure 4A – cluster 1). Indeed, even if no experimental evidence has shown that regulators cannot bind to assembly intermediates yet (Murata et al, 2009), the 19S RP and the PAC1/PAC2 complex are thought to compete for the association with the N-terminal part of the 20S proteasome alpha subunits, through their common HbYX motif (Kusmierczyk et al, 2011). This probably prevents premature associations of nascent alpha rings with activators like the 19S RP (Stadtmueller et al, 2012).**
- **As a last argument, the profile correlations approach aims to cluster proteins having close abundances or sedimentation profiles, depending on the type of experiment (immunopurification and sedimentation gradient fractionation, respectively), independently of the other proteins present in the sample. If the assembly intermediates would have influenced our results, it would have hidden some preferential interactions rather than having created new artefactual ones. Indeed, if the assembly intermediates were too abundant in our experiments, it would have probably flattened the profiles of 20S proteasome subunits and made it more difficult to identify preferential interactions between 20S subtypes and specific regulators.**

Otherwise, their clustering into the selected groups is incorrect.

Our data indeed show that proteasome assembly chaperones (PACs) could be clustered together (Figure 4-B, PCA experiment) but <u>could not be clustered with any of the three protein groups containing 20S proteasome subunits (ncP20S, iP20S, and sP20S) or other regulatory complexes subunits</u>, as shown in Figure 4A and Figure E2A. This information is written in the original manuscript (page 12, line 19): "The PCA also appeared to efficiently cluster the group of Proteasome Assembly Chaperones (PACs 1-4), which could not be correlated with any of the references chosen in the supervised hierarchical clustering statistical method. This might be explained by the fact that these proteins interact with 20S proteasome assembly intermediates and not with matured proteasome forms. ". **So, our data are in agreement with the**

iPBS Institut de Pharmacologie et de Biologie Structurale
www.ipbs.fr
205 route de Narbonne
31077 Toulouse Cedex04
T. 33-5 61 17 59 18
F. 33-5 61 17 59 93
UMR 5089

biological role of these proteins as none of the component of active proteasomes correlates with Proteasome Assembly Chaperones. To clarify Figure 4A, the composition of the clusters has been added in the revised Figure 4A legend. "Three distinct clusters of composition detailed hereafter could be obtained. Cluster 1 (from top to bottom – Figure 4A): Rpt3, Rpn13, α2, Rpn7, USP14, hHR23B, α1, β6, β3, α4, α7, Rpn6, Rpn3, Rpt4, Rpn10, Rpn5, Rpt5, Rpn1, Rpn11, Rpt1, Rpn9, Rpn2, Rpn8, α3, α6, β4, Rpt6, PDC6, Rpn12, APEH, Ubiquilin-1, α5, β7, CCT7, CCT4, CCT2, CCT3, CCT5, CCT6A, DNAJA1, HSP90AB1, HSP90AA1, PNP, Rpt2. Cluster 2 (from top to bottom – Figure 4A): 14-3-3ζ/δ, CAND1, GSR, UBE3C, β2, ATP5A1, ATP5B, β1, PA200, β5, FBXO7, UCHL5, TXNL1, ECM29, PI31. Cluster 3 (from top to bottom – Figure 4A): PA28β, PITH1, β2i, PA28α, PA28γ, β5i, β1i."

iPBS Institut de Pharmacologie et de Biologie Structurale
www.ipbs.fr
205 route de Narbonne
31077 Toulouse Cedex04
T. 33-5 61 17 59 18
F. 33-5 61 17 59 93
UMR 5089

Nevertheless, the presence of assembly chaperones may indicate the frequency of proteasome formation of a certain proteasome type.

As pointed by the reviewer 1, an experimental correlation between some specific chaperones and some specific 20S catalytic subunits would have allowed us to determine if some 20S assembly chaperones were only dedicated to the formation of a particular 20S proteasome subtype. However, as these chaperones cluster with none of the 20S proteasome types, as explained above, our results suggest that none of the five 20S assembly chaperones is specifically involved in the formation of a specific 20S proteasome form. This conclusion was added in the revised manuscript (page 12, line 21):"As these chaperones cluster with none of the 20S proteasome types, our results suggest that none of the five 20S assembly chaperones is specifically involved in the formation of a particular 20S proteasome form."

What does the term ncP20S in this context mean? An active 20S core complex also contains alpha subunits, which are not catalytically active on their own but required as scaffolds in the complex.

- **The ncP20S does not exist as an individual active 20S particle, contrary to the sP20S, the iP20S and the two intermediate 20S proteasomes which correspond to real physical entities, but rather corresponds to a group of non-catalytic subunits (α1- α7 and β3, β4, β6 and β7). Thus, the abundance of the ncP20S corresponds to the abundance of the total 20S proteasome (standard, immuno, intermediate types) because all these 20S proteasome forms contain alpha and non-catalytic beta subunits. Thus, if the abundance of a protein is correlated to the abundance of the ncP20S, it means that this protein is probably associated to all 20S proteasome types, as observed for the 19S regulator. As we agree that the original text was not clear concerning this point, we modified different parts in the revised manuscript:**
    1. **abbreviations section** "ncP20S : non catalytic 20S proteasome subunits (α1- α7, β3, β4, β6, and β7) – representing the total 20S proteasome";

2. **result section: page 10 line 3 "**All 20S proteasome non-catalytic subunits (α1- α7, β3, β4, β6, and β7) (gathered in a group of proteins called "ncP20S")"; **page 11 line 7 "**These were the 19S, PA28αβ, PA28γ, and PA200 activators, the PI31 proteasome regulator, the two major 20S proteasome subtypes, the sP20S (represented by the β5 catalytic subunit) and the iP20S (represented by the β2i catalytic subunit (Guillaume et al, 2010)), and the ncP20S gathering the 20S non-catalytic subunits (thus representing the total 20S proteasome)."; **page 13 line 1** : "Altogether, these results strongly suggest for the first time that the associations between the proteasome 20S subtypes (ncP20S representing total 20S proteasome, sP20S, iP20S),…"; **page 13 lines 22** : "Its abundance is rather correlated with the one of the ncP20S (R^2 of 0.93), which represents all 20S proteasome forms (Figure E2B)."; **page 14 line 19** : "as these proteins exhibit high correlation (R^2 > 0.8) with at least one of the three 20S CP subtypes (total proteasome (ncP20S), sP20S, and iP20S).";
3. **Discussion section:**
   a) **page 19, line 17:** "We arbitrarily called in this study 20S non catalytic proteasome (ncP20S) the subset of the 11 common proteins (α1- α7, β3, β4, β6, and β7) which are present in all 20S proteasome forms; ncP20S thus represents total 20S core particle."
   b) **page 19, line 20:** "A protein that will be associated with the two functional 20S proteasome forms will correlate with subunits of the ncP20S while a protein that will be preferentially associated with the sP20S or with the iP20S will correlate with the β5 subunit or with the β2i subunit, respectively.".

iPBS
Institut de Pharmacologie et de Biologie Structurale
www.ipbs.fr
205 route de Narbonne
31077 Toulouse Cedex04
T. 33-5 61 17 59 18
F. 33-5 61 17 59 93
UMR 5089

The authors ignore the existence of so-called hybrid proteasomes, in which the 20S core complex (most likely immunoproteasome 20S) is associated with the 19S regulator on one side and with the 11S regulator (PA28 a, b) on the other (Tanahashi N et al., JBC 2000). Can the authors conclude from their correlations whether hybrid proteasomes exist as one entity in their preparations? Here, a supportive biochemical control experiment (native PAGE analyses and immunoblotting stained for PA28 and a 19S subunit or immunoprecipitation and counterstaining for both) should be performed to clarify this.

We agree that hybrid proteasomes are indeed important proteasome assemblies that certainly need to be considered. A reference to hybrid proteasomes has been added in the introduction (page 5, line 4): "One 20S CP can interact at its two sides with either two identical regulators or two different ones, thus forming hybrid proteasomes (Tanahashi et al, 2000).").

The correlations data cannot conclude whether hybrid proteasomes exist as one entity in the preparations because, to our knowledge, there is no protein that is specific of hybrid proteasomes.

However, the glycerol gradient sedimentation experiment associated with the detection by mass spectrometry is equivalent to the experiment proposed by the reviewer to answer this point (native PAGE analyses and immunoblotting stained for PA28 and a 19S subunit). We have indeed experienced bad-quality results using native PAGE, probably on account of the difficulty of formaldehyde cross-linked protein complexes

www.ipbs.fr
205 route de Narbonne
31077 Toulouse Cedex04
T. 33-5 61 17 59 18
F. 33-5 61 17 59 93
UMR 5089

to run into the polyacrylamide gel. Tanahashi *et al.* (Tanahashi et al, 2000) have also used glycerol density gradient centrifugation associated with immunodetection to analyse proteasome heterogeneity. After sedimentation, all the glycerol gradient fractions containing active proteasome complexes were analyzed by mass spectrometry and all subunits of the 19S, PA28αβ, and PA28γ RPs could be quantified (which is probably superior to immunoblotting because all the 19S RP's subunits can be quantified in a single analysis). Importantly, to allow a precise quantification, the MS inter-run signal intensities variations were handled thanks to a normalization using isotopically labelled peptides (AQUA peptides). These peptides were introduced in the different trypsin-digested protein fractions just before injection on the nanoLC MS/MS system (as detailed in the Material & Methods section). As shown in the Figure below where proteasome complexes from U937 cells were separated according to their different sedimentation coefficients, fraction 16 (20% glycerol) contains mainly free 20S proteasome (the intensity of the 20S Proteasome's peak shows that the proportion of free 20SP is high, confirming our previous work (Fabre et al, 2013)) while co-eluting peaks of 19S RP subunits and 20S subunits (with no detection of PA28αβ nor PA28γ RPs) are clearly identified in fraction 7 (32% glycerol), showing here the presence of 30S proteasome. Fractions 12 to 15 (22 to 26% glycerol) probably contain 20S proteasome associated with one PA28 RP (PA28αβ or PA28γ) and Fractions 11/12 (26 – 27 % glycerol) probably contain 20S CP associated with either two PA28 RPs or with one 19S RP. Very interestingly, intense and co-eluting peaks of 20S, 19S, and both PA28 RPs (PA28αβ and PA28γ) are detected in fraction 10 (28% glycerol), strongly suggesting the presence of hybrid proteasomes.

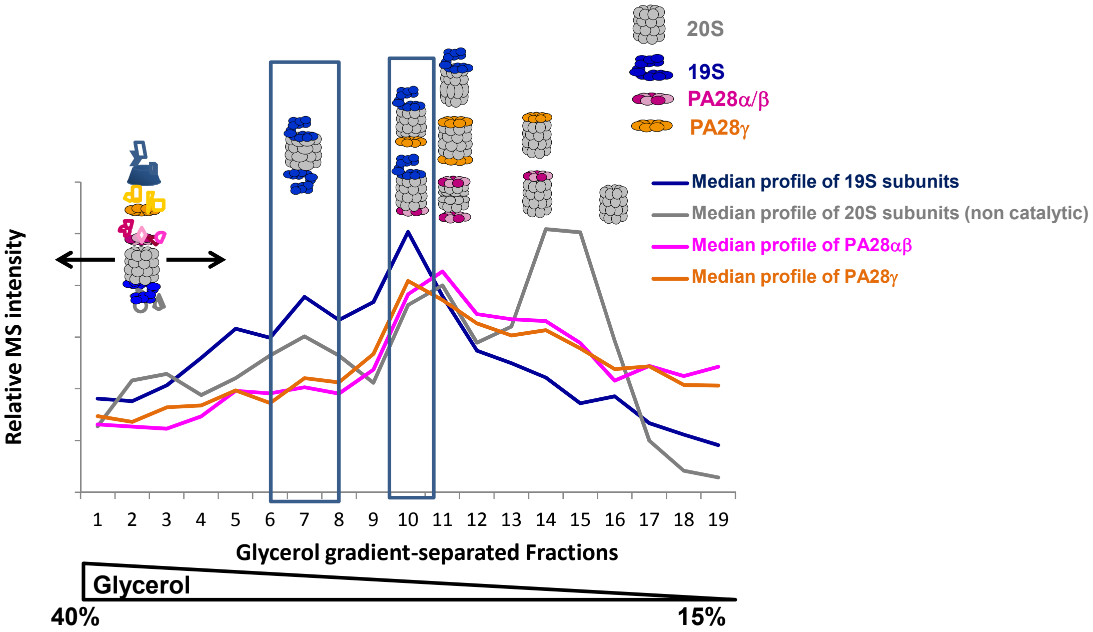

An additional immunopurification experiment using an antibody directed against the PA28β protein (Cell Signaling – Ref 2409) was performed together with a control consisting of an immunopurification

using antibodies from pre-immunized rabbits (*n*=2). U937 cells were chosen because they contain high levels of 20S associated-PA28αβ regulator. Immunopurified PA28β, PA28α, and 19S subunits were then quantified by quantitative MS (LTQ-Orbitrap Velos Instrument, Thermo), as shown below:

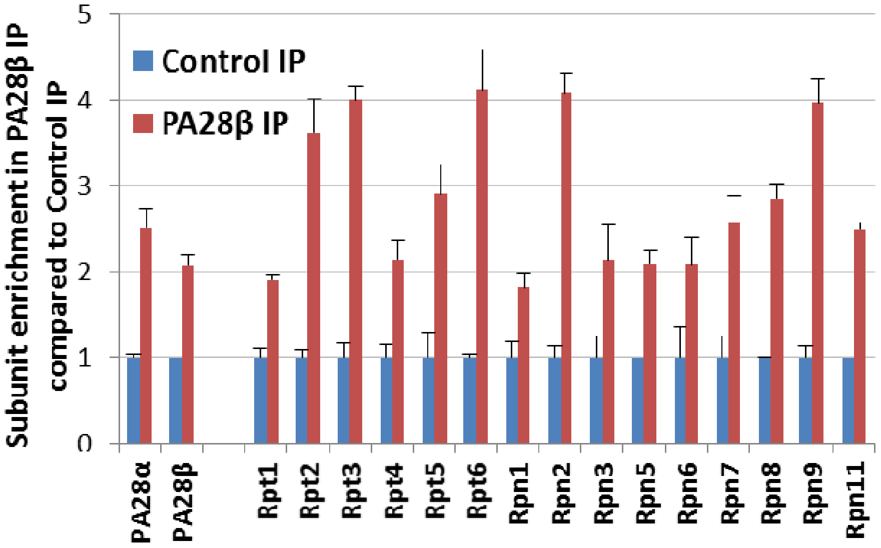

PA28α and PA28β subunits, but also 19S subunits are all enriched in the immunopurification compare to the control, indeed confirming the existence of hybrid proteasomes in U937 cells.

Hybrid proteasomes have been described in various mammalian cells (Tanahashi et al, 2000), so they are also expected to be present in all the human cell lines studied here. The correlations of the abundances of 19S subunits and PA28α/PA28β proteins is rather low (mean r^2 of 0.42 ± 0.09) which is explained by the fact that 19S and PA28αβ RPs are involved in several different types of functional proteasome complexes (30S, 26S, for 19 RP; 20S- PA28αβ, PA28αβ-20S-PA28αβ for PA28αβ RP) in addition to hybrid proteasomes. This was added in the result section of the revised manuscript (page 10, line 11): "Conversely, when plotting the PAIs of Rpn3 and PA28β, proteins belonging to the 19S RP and the PA28αβ regulator, respectively, a much weaker correlation (R^2 = 0.44) could be observed (Figure E3). This is probably because the 19S RP is involved in several different types of functional proteasome complexes (30S, 26S for instance) in addition to hybrid proteasome (one 19S RP and one PA28 RP associated with one 20S CP), which could indeed be observed in U937 cells when immuno-purifying PA28β (Figure E1C)." (only the underlined part is new in the manuscript).

iPBS
Institut de Pharmacologie et de Biologie Structurale
www.ipbs.fr
205 route de Narbonne
31077 Toulouse Cedex04
T. 33-5 61 17 59 18
F. 33-5 61 17 59 93
UMR 5089

iPBS
Institut de Pharmacologie et de Biologie Structurale
www.ipbs.fr
205 route de Narbonne
31077 Toulouse Cedex04
T. 33-5 61 17 59 18
F. 33-5 61 17 59 93
UMR 5089

This reviewer also wonders why the authors do not refer to their own work on intermediate type proteasomes (containing standard as well as immuno-active sites)? Is the method used suitable to detect this subtype? These proteasomes also contribute to the heterogeneity and should be mentioned and at least discussed in this context.

Two intermediate proteasomes have been isolated by Guillaume *et al.* (Guillaume et al, 2010) in addition to the standard and immuno 20S proteasomes. As explained in the introduction of the revised manuscript (line 16-18, page 4), the β5i intermediate 20S proteasome (β5i P20S) contains the β1 and β2 standard subunits together with the β5i immunosubunit. The β1i β5i intermediate 20S proteasome (β1iβ5i P20S) contains the β2 standard subunit and the β1i and β5i immunosubunits. As shown earlier by Guillaume *et al.* (Guillaume et al, 2010), many tissues and cell lines exhibit varying amounts of the four 20S proteasome subtypes. This is also the case of the various cell lines studied here, as we published in an earlier work (Fabre et al, 2014). However, contrary to standard and immuno 20S proteasomes which both contain a catalytic subunit that is specific to each of them (β5 is specific to the standard 20S proteasome and β2i to the immuno 20S proteasome), the β5i and β1i β5i intermediate 20S proteasomes do not display any specific subunit and have very similar MW as standard and immuno P20S. Thus, it is much more difficult to detect proteins associating specifically with these two intermediate subtypes when all 20S proteasome subtypes are present, as observed *in vivo*. However, we could purify proteasomes from HEK cell lines transfected with either β5i or with β1i and β5i (and therefore expressing a unique intermediate P20S, β5i P20S or β1i β5i P20S, respectively), as published in Guillaume *et al.* (Guillaume et al, 2010). In these two cell lines, we observe that these two intermediate 20S proteasomes associate with the same main P20S regulators as the immunoproteasome (iP20S) (Figure E6B). This expanded figure (Figure E6 A & B) and a mention to these two intermediate 20S subtypes have been added in the revised version of the manuscript (Page 15, line 19): "Interestingly, proteasome immunopurified from HEK EBNA cells transfected with either β5i alone or with β1i and β5i (Figure E6A) (and therefore expressing a unique intermediate P20S, β5i P20S or β1iβ5i P20S, respectively (Guillaume et al, 2010)) associate with the same main P20S proteasome regulators as the immunoproteasome (Figure E6B).").

The experiments in Figure 5 are nice to support the drawn conclusions from the first more systems biology approach. However, to draw these conclusions an expression control (immunoblot) for representative regulator subunits is required (PA28, 19S).

The expression levels of the different P20S subtypes (ncP20S, iP20S, sP20S) as well as the ones of the major 20S regulators (PA28αβ, PA200, PA28γ, and PI31) were measured using quantitative mass spectrometry analysis of the total HeLa cell lysates during the stimulation with IFNγ. Protein complexes abundances were normalized with the abundance of

histones (as loading control, similarly as it would be done by immunoblot).

As shown in Figure E7B, only sP20S, iP20S and PA28αβ are significantly changed (by factors of 0.2, 19, and 4.5, respectively). The cellular levels of all the other P20S regulators (PA28γ, PA200, 19S, PI31) are not significantly modified in the cell lysates and during the cytokine stimulation. Thus, the differential interactions observed in the proteasome immunoprecipitates, in particular the ones involving PI31 and PA200, are not caused by changes of the expression of these proteins. This expression control has been added as Figure E7B and the manuscript has been revised to include it in the text: page 16, line 17: "These differential interactions, in particular the ones involving PI31 and PA200, are not caused by changes of the expression of these proteins in the total lysates of the IFNγ-stimulated HeLa cells (Figure E7B – Expanded table 7)".

iPBS
Institut de Pharmacologie et de Biologie Structurale
www.ipbs.fr
205 route de Narbonne
31077 Toulouse Cedex04
T. 33-5 61 17 59 18
F. 33-5 61 17 59 93
UMR 5089

Why the authors did not use beta5i as the immunoproteasome counterpart of beta5?

A recent study has shown that only four conformations of 20S proteasome can be isolated in human cells (Guillaume et al, 2010). These four human 20S proteasome subtypes differ by their composition in catalytic subunits: standard P20S (β1, β2, β5), immuno P20S (β1i, β2i, β5i), β5i intermediate P20S (β1, β2, β5i), and β1iβ5i intermediate P20S (β1i, β2, β5i). Thus, though β5i replaces β5 in the imunoproteasome and therefore constitutes the β5's immuno counterpart, β5i is present in three 20S subtypes (iP20S, β5i P20S, and β1iβ5i P20S) (Figure E6-A). Contrary to β5i, β2i is the only subunit that is specific of the iP20S (Figure E6-A). Therefore, β2i's abundance and not β5i's is used to quantify iP20S. That is the reason why we use the abundances of β5 and β2i to compare the abundances of standard proteasome and immunoproteasome, respectively.

Minor points

The discussion is too long (6 pages for 5 Figures).

We shortened the discussion to 5 pages:

- **The analysis of the work of Kim *et al* 2014 (Kim et al, 2014) was placed in the result section, as suggested by reviewer 3.**
- **The concluding remarks were skipped because they were redundant with the abstract.**
- **Three other sentences were also skipped in the discussion:**
    1. **Page 21:** "Interestingly, proteasome immunosubunits as well as PA28α and PA28β genes are all expressed during early mouse embryogenesis (Hamatani et al, 2004)."
    2. **Page 21:** "In the nine cell lines studied, we previously showed that fully assembled proteasomes represent more than 85% of the total proteasome

pool (Fabre et al, 2014). Therefore, we speculate that the functional role of PI31 is linked to mature sP20S."

Sometimes the references are a bit strange and not up to date. As an example there are very nice structural studies of human 26S proteasomes by the Morris group (da Fonseca et al., Mol Cell 2012) showing the gate opening and the specific interaction sites.

References have been carefully reviewed.

The following references have been added:

- **Tanahashi N. J. Biol. Chem, 2000 (page 5) (as suggested by the reviewer)**
- **Basler, Kirk et al, Curr Opin Immunol, 2013 (pages 4 & 21)**
- **Beck et al., PNAS, 2012 (page 5)**
- **Da Fonseca et al., Mol Cell 2012 (page 5) (as suggested by the reviewer)**
- **Lander et al., Nature, 2012 (page 5)**
- **Lasker et al., PNAS, 2012 (page 5)**
- **Gatto, Vizcaíno et al, Proteomics, 2010 (page 6)**
- **Cascio P, Biomolecules, 2014 (page 21)**
- **Raule, Cerruti et al, Chem Biol, 2014 (page 21)**
-

The following references have been skipped (too old or not initially well-chosen):

- **Shimbara, N., H. Nakajima, et al., Genes Cells, 1997 (page 21)**
- **Kusmierczyk, Kunjappu et al. 2011, (page 22)**

A better explanation for the content of the supplemental tables would be helpful for readers.

A new worksheet (placed in the first position and entitled "Explanation_content") has been added in all supplemental tables (excel files).

iPBS
Institut de Pharmacologie et de Biologie Structurale
www.ipbs.fr
205 route de Narbonne
31077 Toulouse Cedex04
T. 33-5 61 17 59 18
F. 33-5 61 17 59 93
UMR 5089

Reviewer #2:

This manuscript describes a combination of AP-MS and PCP to characterize the normal and immunoproteasomes from many different cell lines to look at the differences in stoichiometry of attached regulatory molecules. It is nicely done presents a compelling story, although there is not really any mechanisms being tests. That may not be essential though, in this case.

Concerns

- I am concerned that the high correlation described for Figure 3 may be an artefact of the immunopurification approach used. The details of the IP conditions do not seem to be described in the Methods for one thing but if the same antibody is used for each cell line (as I presume it was) then perhaps the antibody can only pull down certain configurations of the proteasome that would have similar abundances of their subunits? This could be addressed with alternative antibodies or by comparison to a different method, such as PCP.

Possible artefactual correlations possibly induced by the MCP21 antibody used for proteasomes immunopurification indeed constitute an important concern that needs to be addressed.

The MCP21, which targets the alpha 2 subunit of the 20S proteasome), was indeed chosen as a unique antibody for proteasomes immunopurification for several reasons:

- **First, it belongs to the 20S core particle and it is a constitutive subunit; it is therefore supposed to be present in all existing subtypes of 20S particles and should, in principle, enable to capture all cellular proteasome complexes. Catalytic subunits, which exist in two different forms (standard or immuno-subunits), do not however constitute appropriate targets.**
- **Second, the MCP21 antibody recognizes efficiently the alpha2 subunit, even when the target subunit is assembled in the physiological complex (epitope still recognized by the antibody). Any other constitutive subunit of the 20S core might have been used as a prey as far as the epitope is not hindered when the physiological complex is assembled. As a routine control to confirm this, we analyze the total lysate and the supernatant after IP and compare the proteasome chymotrypsin-like activity in the total lysate and in the supernatant after IP: as shown below, yields over 80% can be observed in all cell lines (87 ± 5 %). Remaining but still low chymotrypsin activity can be measured in the supernatant after IP on account of the presence of other proteases than proteasome.**

iPBS
Institut de Pharmacologie et de Biologie Structurale
www.ipbs.fr
205 route de Narbonne
31077 Toulouse Cedex04
T. 33-5 61 17 59 18
F. 33-5 61 17 59 93
UMR 5089

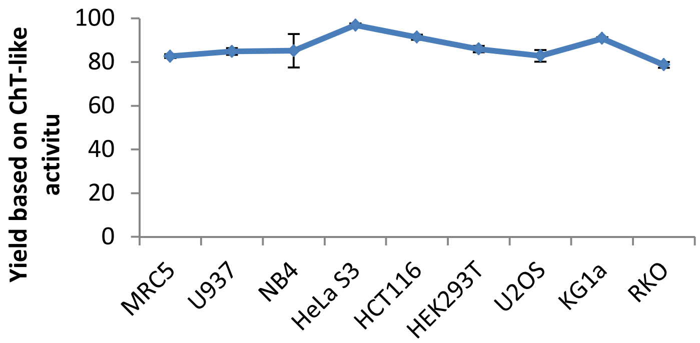

In some cases, as another control, we also perform immunodetection analyses of 20S subunits (using either the MCP21 antibody or the commercial antibody from Enzo Life Sciences directed against core alpha subunits of the 20S proteasome Ref: BML-PW8155) in the total lysate ("Input") and in the flow-through after IP ("FT"). Below is an example obtained for U937, KG1a, and HeLa cells:

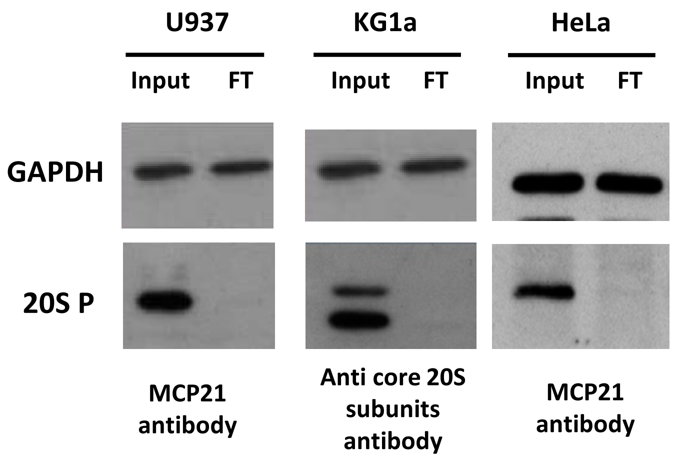

Similar results would be expected with proteasomes immunopurification using an antibody directed against another alpha subunit like the alpha 3 subunit for example which has been used to purify the 26S proteasome from mice heart lysates (Drews et al, 2010). Moreover, a recent study in which antibodies directed against different proteasome subunits in ChIP experiments to look at proteasome complexes associated to the transcription machinery showed very similar results when using antibodies against two different alpha subunits (Geng & Tansey, 2012).

- Third, the quantities of antibody required to allow such studies (enzymatic activity measurements and proteasomes purification, identification and relative quantification), are quite important (in the order of mg). We thus used a hybridoma cell line to produce the anti-α2 antibody (MCP21) and to reduce costs.
Other hybridoma clones against α4 (clone MCP34), α6 (clone MCP20), α3 (clone MCP102) or α7 (clone MCP444) might also be used as far as the produced monoclonal antibody still recognizes its target when assembled in the endogeneous complex. So, the use of another 20S proteasome subunit as a target might also be considered as far as it meets the above requirements.

iPBS Institut de Pharmacologie et de Biologie Structurale
www.ipbs.fr
205 route de Narbonne
31077 Toulouse Cedex04
T. 33-5 61 17 59 18
F. 33-5 61 17 59 93
UMR 5089

iPBS
Institut de Pharmacologie et de Biologie Structurale
www.ipbs.fr
205 route de Narbonne
31077 Toulouse Cedex04
T. 33-5 61 17 59 18
F. 33-5 61 17 59 93
UMR 5089

Finally and as a last argument to convince the reviewer, the PCP analysis performed on the glycerol gradient-sedimented proteasomes (Figures 1 & 2) concludes to a correlation between the iP20S and the PA28αβ RP, as does the correlation analysis in the proteasome immunoprecipitates (purified with the MCP21 antibody). Yet, the PCP analysis on the sedimented proteasomes is completely independent (no immunopurification performed at all) from the experiment where proteasomes were immunopurified using the MCP21 antibody. So, if an artefact would arise from the immunopurification with the MCP21 antibody, we sincerely think that the correlation between PA28αβ and iP20S would not be observed in the PCP/sedimentation analysis.

Modifications made in the revised manuscript:

- **A more detailed description of the immunopurification of proteasomes using the MCP21 antibody was added in the revised manuscript (page 23):** "Briefly, each cell lysate sample was incubated with 100mg of CNBr sepharose beads (GE Healthcare) coupled with 0.8mg MCP21 antibody (directed against the α2 subunit of the 20S proteasome). Supernatants (S1) were collected and beads were washed three times with 40 bed volumes of washing buffer (20mM Tris-HCl pH 8, 1mM EDTA, 10% glycerol, 150mM NaCl, 0.1% NP40, 10mM ATP and 2mM MgCl2) and proteins were eluted with 0.5 mL of elution buffer (20mM Tris-HCl pH 8, 1mM EDTA, 10% glycerol, 3M NaCl, 10mM ATP and 2mM MgCl2). Beads were then washed and incubated with supernatants S1. Two additional cycles of purification were repeated and the three eluates were finally pooled together.".
- **A reference to the purification yield was added in the text (page 9 line 15:** "Very high 20S proteasome purification yields (87 ± 5 %) could be obtained (Figure E1A). ".) **and Figure E1A was added.**

- Mechanisms: I am not a proteasome expert but it would seem that an extension of this work could be in vitro assays of proteasome function are tested with the various combinations of regulatory proteins identified here. I appreciate though that such assays are not yet available, at least where additional individual components can be added or subtracted.

To answer to this reviewer concern, we performed several *in vitro* experiments as detailed below:

1. **<u>Interaction between 20S proteasome and PI31 regulator:</u> a commercially-available N-terminus His-tagged form of human PI31 (produced in *E coli* – Novus Biologicals ref NBP1-78851) was incubated with equal molar amounts of fully-assembled sP20S and iP20S (produced in-house) during 15min, 1h, and overnight (molar ratio of PI31/20S proteasome equal to 41). PI31 was purified using an antibody directed against the His-tag and the co-purified proteasome was then analyzed by mass spectrometry (MRM assay). The molar ratio of β5 over β2i was calculated during the kinetics in the control (no PI31 added) and in the assay to measure a relative enrichment of sP20S over**

iP20S in the assay (“A”) compared to the control (“C”) (*n*=3). The results shown below indicate that sP20S is enriched by a factor of 3.7 ± 0.1 compared to the iP20S at 15 min of incubation. After an overnight incubation, this ratio is decreased to 2.9 ± 0.1 probably because PI31 is in molar excess compared to total 20S proteasome (41 fold molar excess). These results thus support our conclusion that PI31 interacts preferentially with the sP20S.

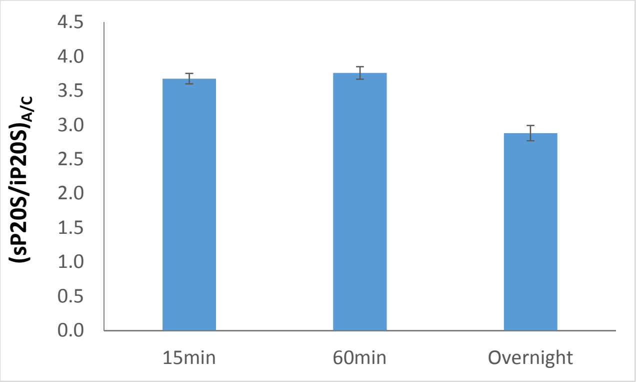

iPBS Institut de Pharmacologie et de Biologie Structurale
www.ipbs.fr
205 route de Narbonne
31077 Toulouse Cedex04
T. 33-5 61 17 59 18
F. 33-5 61 17 59 93
UMR 5089

2. <u>Interaction between 20S proteasome and PA28αβ regulator</u>:

a) The same experiment as the one described above was performed with a commercially-available His-tagged form of human PA28β (recombinant protein produced in *E. coli* - Boston Biochem ref E-382) (incubation with equal molar amounts of fully-assembled sP20S and iP20S during 15min, 1h, and ON - PA28β introduced in 42 fold molar excess compared to total P20S - and measurement of β2i over β5 ratio after purification of PA28β using an antibody directed against the His-tag). No enrichment of the iP20S over the sP20S could be observed in this case.

b) Another *in vitro* affinity-purification assay was performed using an endogenous PA28αβ complex (purified from human erythrocytes; ENZO Ref PW9420 – able to activate 20S proteasome, as measured using our proteasome activity assay) and an antibody directed against PA28β (Cell Signaling Ref 2409). The purified PA28αβ complex was first incubated with equal molar amounts of fully-assembled sP20S and iP20S (produced in-house) during 15min, 1h, and ON (molar ratio of PA28αβ/total 20S proteasome equal to 7). A control experiment was performed with no PA28αβ complex added. Then, an affinity purification using the anti PA28β antibody was performed and the purified proteins were quantified by MRM. The molar ratio of β2i over β5 was calculated during the kinetics in the control and in the assay to measure a relative enrichment of iP20S over sP20S in the assay (“A”) compared to the control (“C”) (*n*=3). The results shown below indicate that iP20S is enriched by a factor of 1.58 ± 0.19 compared to the sP20S at 15 min of incubation. After an overnight incubation, this ratio is decreased to 1.15 ± 0.16. The *in vitro* preferential interaction of PA28αβ with iP20S compared to sP20S is thus observable but much more limited than *in vivo*.

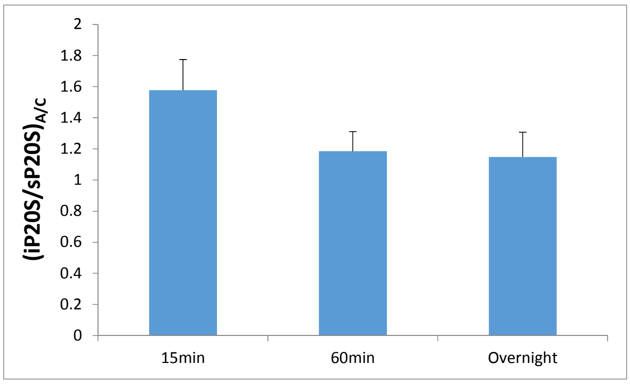

iPBS
Institut de Pharmacologie et de Biologie Structurale
www.ipbs.fr
205 route de Narbonne
31077 Toulouse Cedex04
T. 33-5 61 17 59 18
F. 33-5 61 17 59 93
UMR 5089

Several reasons can explain that the *in vitro* assays do not fully agree with our *in vivo* observations.

- **First, the monomeric His-tagged PA28β protein might not multimerize and recognize 20S proteasome, as does the native PA28αβ heptamer.**
- **Second, the proteins used are either recombinant proteins (produced in bacterial system – His-tagged PA28β), or purified from human erythrocytes (containing almost exclusively standard proteasome). They thus might not contain essential PTM or protein variant required for preferential association with a specific 20S subtype.**
- **Third, other cellular proteins/factor, absent *in vitro*, are likely to trigger the *in vivo* observed preferential interactions between 20S subtypes and specific interactors explaining that it cannot be observed *in vitro*. This phenomenon has been already reported for the interaction between the 26S proteasome and polyubiquitin chains. The Goldberg group has indeed elegantly demonstrated that though pure 26S proteasome associates and degrades similarly K48- and K63- ubiquitin substrates *in vitro*, cellular soluble factors mediates the preferential degradation of K48 polyubiquitinated substrates *in vivo* (Nathan et al, 2013).**

For these reasons, we sincerely think that our *in vivo* results obtained either with cell lines models expressing unique 20S subtypes or by physiologically changing the 20S proteasome composition (results displayed in Figure 5) are much more relevant than *in vitro* studies. Therefore, these results were not included in the manuscript.

The detailed protocols can be sent, if the reviewer feels it necessary.

- I'm not sure that the field needs another acronym, especially one as hard to remember as "PAC-AP-HR-MS". Since this is not a methods paper, I suggest aspect of it just be removed since it doesn't really add anything. In addition, focusing on the High Resolution aspect of the MS seems a little passé since virtually all proteomics is done with high res instruments now (Orbitraps, QTOFs). Gone (thankfully) are the days when 3D or 2D traps dominated!

The acronym "PAC-AP-HR-MS" as well as the term "HR" were removed.

Reviewer #3

Fabre et al investigate proteins associated with the proteasome and immunoproteasome by mass spectrometric techniques. Specifically, they combine correlation profiling, where successive fractions are analyzed by quantitative MS to obtain characteristic profiles, with pulldowns, a technique which they call "Protein Abundance Correlation associated to Affinity Purification and High Resolution Mass Spectrometry" (note that this would be called 'associated with' to be grammatically correct).

"Associated to" has been replaced by "associated with" in the revised manuscript

Overall, the ideas and the results are quite interesting. The major shortcoming is that this technique is demonstrated on the proteasome, which is extremely abundant and one of the easiest complexes to work on. It is not clear that there would be many other complexes where this technique would work.

We have not tried the technique on other complexes yet but the affinity purification approach coupled to mass spectrometry (AP-MS) is one of the most efficient strategy to enrich a low-abundance complex and to determine its associated partner (Dunham et al, 2012; Nagore et al, 2013). Many low-abundance protein complexes have been successfully characterized using AP-MS such as protein kinases or other networks (Breitkreutz et al, 2010; Couzens et al, 2013; Malovannaya et al, 2011). Tagging strategies simplify the purification procedure of low-abundance complexes when no antibody is available. We believe that the existing data could be reanalyzed using the described strategy to possibly determine preferential interactions within subcomplexes of published protein assemblies. Alternatively, affinity-purified samples could be analyzed using targeted MS approaches (like Single Reaction Monitoring or SWATH-MS) to increase the sensitivity and the quantification accuracy (Gallien et al, 2011; Picotti & Aebersold, 2012; Picotti et al, 2013).

Furthermore, while the combination of approaches is novel, none of the individual parts are. Therefore a big part of the novelty would have to be in the new associations of factors to the proteasome and immunoproteasome. Of course, it would have been nice if these novel associations had been tested by independent means, but I realize that this may be difficult.

The two strategies described in the two parts of the work (Protein Correlation Profiling analysis on glycerol gradient-sedimented proteasomes and correlations of abundances on immunopurified proteasomes) constitute independent approaches and both of these lead to the same conclusion that the immunoproteasome preferentially associates to the PA28αβ regulator. Moreover, the IFNγ-mediated modulation of standard and immunoproteasomes levels and their associating regulators

iPBS
Institut de Pharmacologie et de Biologie Structurale
www.ipbs.fr
205 route de Narbonne
31077 Toulouse Cedex04
T. 33-5 61 17 59 18
F. 33-5 61 17 59 93
UMR 5089

corroborates the data obtained with the above two approaches and constitutes an *in vivo* physiological asset of the conclusions.

Specific points:
The paper seems to excessively long and this itself makes the logic difficult to follow. As an example, one doesn't need to summarize the findings at the end of the introduction so extensively.

The end of the introduction was shortened as well as the discussion, as requested by reviewer 1.

iPBS
Institut de Pharmacologie et de Biologie Structurale
www.ipbs.fr
205 route de Narbonne
31077 Toulouse Cedex04
T. 33-5 61 17 59 18
F. 33-5 61 17 59 93
UMR 5089

Use of formaldehyde cross-linking before protein correlation profiling is interesting. Can the authors compare results with and without doing this, so we know what factors are retained only due to the crosslinking? (Maybe this is described in their Fabre 2013 or 2014 paper, but most readers would not look this up.)

The benefit of formaldehyde cross-linking on Proteasome Interacting proteins (PIPs) recovery has indeed been specifically studied in previous papers (Bousquet-Dubouch et al, 2009; Fabre et al, 2013). For instance, in human erythrocytes, formaldehyde treatment enabled important PIPs like ECM29, HSP90β, and multiubiquitin chain-binding proteins (hHR23B, DDI1 homolog 2, and eEF1α1) to be retained with proteasomes or greatly increased the recovery of others like USP14 (an important deubiquitinylase associated with proteasomes) (Bousquet-Dubouch et al, 2009). In our more recent paper published in 2013 (Fabre et al, 2013), we showed that a low formaldehyde concentration (0.1%, the same concentration as the one we have used in the presently submitted work) could help to dramatically stabilize the association of all regulatory particles to the 20S core particle. As pointed by the reviewer, this stabilization of proteasome complexes induced thanks to formadehyde cross-linking is very important for the protein correlation profiling analysis because this approach is based on quantitative data. Thus, a reference to the stabilizing effect of formaldehyde was added in the revised manuscript (page 9, line 10: "Formaldehyde cross-linking was shown to be required to stabilize the association of all regulatory particles with the 20S core particle (Fabre et al, 2013)."**).**

People usually use just the term R2 instead of 'coefficient of determination'

The term "coefficient of determination" has been skipped and replaced by the term "R^2" except when this coefficient was defined the first time.

The authors mention in the discussion that most PIPs were not identified in this study 'probably because of the low fractionation level used'. This is a pity and seems to contradict what the authors say in the beginning of the results where they have 120 PIPs. An alternative explanation would be that many of the previously reported PIPs were actually artifacts. If the technique introduced here is more comprehensive as the authors claim, could it not shed light on which previously reported PIPs are probably spurious?

iPBS
Institut de Pharmacologie et de Biologie Structurale

www.ipbs.fr

205 route de Narbonne
31077 Toulouse Cedex04

T. 33-5 61 17 59 18
F. 33-5 61 17 59 93

UMR 5089

It seems that the reviewer misunderstood what we meant by « In the present study, only the most abundant proteasome regulators like 19S, PA28αβ, or PA28γ could be identified in the 19 glycerol gradient fractions analyzed, probably because of the low fractionation level used. Most of the other known PIPs were missing, probably because of their sub-stoichiometric integration into proteasomes. As an alternative to extensive fractionation, we combined the strengths of AP-MS and PCP-HR-MS to comprehensively analyze proteasome complexes heterogeneity."

This paragraph indeed refers to the result part where we compare the number of identified PIPs using glycerol gradient separation and PCP- MS (1st paragraph of the results section entitled "*PCP- MS analysis of glycerol density gradient-separated proteasome complexes*") (73 PIPs identified) with the number of identified PIPs using the new approach (coupling of AP-MS and PCP- MS) (3rd paragraph of the results section entitled "*Proteasome subunits and associated proteins cluster differently on the basis of their abundances across the nine cell lines*") (120 PIPs identified): "Coupling AP-MS to protein correlation profiling also allowed to more comprehensively characterize proteasome heterogeneity because a much higher number of known proteasome interacting proteins (PIPs) were identified (120 PIPs) as compared to the initial glycerol gradient analysis (where 73 PIPs were identified)" **(page 13, line 5).**

From this result, we deduced that the PCP-MS analysis on glycerol-gradient fractionated proteasomes could not permit to identify as many PIPs as the new approach developed (combination of AP-MS and PCP-MS). So, to allow a better understanding, the sentences in the discussion have been rephrased as (page 18, line 15): "The PCP-MS analysis we performed on U937 AML cells proteins separated by glycerol density gradient ultracentrifugation enabled to reveal for the first time a previously unreported preferential between iP20S and PA28αβ RP. However, in the 19 glycerol gradient fractions analyzed, only the most abundant proteasome regulators like 19S, PA28αβ, or PA28γ could be identified, probably because of the low fractionation level used. Many of the other known PIPs were missing, probably because of their sub-stoichiometric integration into proteasomes. So, as an alternative to extensive fractionation, we developed a new workflow combining the strengths of AP-MS and PCP-MS to significantly increase the number of PIPs identified and thus to comprehensively analyze proteasome complexes heterogeneity.".

To finish, as written in the original manuscript (page 11, line 12: "Of the 170 human Proteasome Interacting Proteins identified in previous AP-MS experiments (Andersen et al, 2009; Bousquet-Dubouch et al, 2009; Wang & Huang, 2008), 120 have been quantified in this survey and, among these, 70 proteins exhibited a high correlation (R > 0.8) with at least one of the references, suggesting that these proteins constitute reliable proteasome partners."), **our technique indeed allows to confirm reliable PIPs but cannot claim that the other PIPs identified in the other published works are not real partners. Proteasomes constitute very dynamic assemblies and interacting proteins are certainly changed with cell types, physiological context, drug treatment (Kaake et al, 2014).**

If the authors want to use the results of the Kim et al. 2014 study, this should be folded into the results section.

The analysis obtained from the Kim et al. 2014 study was folded in the result section.

Figures would look nicer in color (i.e. figure 1).

Figures were colored

Check grammar and spelling in the manuscript (i.e. Protein Abondance Index in figure 3).

Also the PAI paper is not cited.

The authors use comma "," where they should use period "." to denote the decimal point.

- **We checked that periods were used to denote the decimal point.**
- **The label-free quantitative method used in this work is the TOP3 approach initially described by Silva *et al.* (Silva et al, 2006) (see page 7 line 18: "**Label free quantification based on peptide ion extracted chromatograms was performed (Gautier et al, 2012; Mouton-Barbosa et al, 2010) using the TOP3 quantification method (Silva et al, 2006). A Protein Abundance Index (PAI) was calculated to approximate the relative quantity of each proteasome subunit and proteasome-associated protein."**).**

iPBS
Institut de Pharmacologie et de Biologie Structurale
www.ipbs.fr
205 route de Narbonne
31077 Toulouse Cedex04
T. 33-5 61 17 59 18
F. 33-5 61 17 59 93
UMR 5089

References

Andersen KM, Madsen L, Prag S, Johnsen AH, Semple CA, Hendil KB, Hartmann-Petersen R (2009) Thioredoxin Txnl1/TRP32 is a redox-active cofactor of the 26 S proteasome. *The Journal of biological chemistry* **284:** 15246-15254

Bousquet-Dubouch MP, Baudelet E, Guerin F, Matondo M, Uttenweiler-Joseph S, Burlet-Schiltz O, Monsarrat B (2009) Affinity purification strategy to capture human endogenous proteasome complexes diversity and to identify proteasome-interacting proteins. *Mol Cell Proteomics* **8:** 1150-1164

Breitkreutz A, Choi H, Sharom JR, Boucher L, Neduva V, Larsen B, Lin ZY, Breitkreutz BJ, Stark C, Liu G, Ahn J, Dewar-Darch D, Reguly T, Tang X, Almeida R, Qin ZS, Pawson T, Gingras AC, Nesvizhskii AI, Tyers M (2010) A global protein kinase and phosphatase interaction network in yeast. *Science* **328:** 1043-1046

Couzens AL, Knight JD, Kean MJ, Teo G, Weiss A, Dunham WH, Lin ZY, Bagshaw RD, Sicheri F, Pawson T, Wrana JL, Choi H, Gingras AC (2013) Protein interaction network of the mammalian Hippo pathway reveals mechanisms of kinase-phosphatase interactions. *Science signaling* **6:** rs15

Drews O, Tsukamoto O, Liem D, Streicher J, Wang Y, Ping P (2010) Differential regulation of proteasome function in isoproterenol-induced cardiac hypertrophy. *Circ Res* **107:** 1094-1101

Dunham WH, Mullin M, Gingras AC (2012) Affinity-purification coupled to mass spectrometry: basic principles and strategies. *Proteomics* **12:** 1576-1590

Fabre B, Lambour T, Delobel J, Amalric F, Monsarrat B, Burlet-Schiltz O, Bousquet-Dubouch MP (2013) Subcellular distribution and dynamics of active proteasome complexes unraveled by a workflow combining in vivo complex cross-linking and quantitative proteomics. *Mol Cell Proteomics* **12:** 687-699

Fabre B, Lambour T, Garrigues L, Ducoux-Petit M, Amalric F, Monsarrat B, Burlet-Schiltz O, Bousquet-Dubouch MP (2014) Label-free quantitative proteomics reveals the dynamics of proteasome complexes composition and stoichiometry in a wide range of human cell lines. *Journal of proteome research* **13:** 3027-3037

Gallien S, Duriez E, Domon B (2011) Selected reaction monitoring applied to proteomics. *J Mass Spectrom* **46:** 298-312

Gautier V, Mouton-Barbosa E, Bouyssie D, Delcourt N, Beau M, Girard JP, Cayrol C, Burlet-Schiltz O, Monsarrat B, Gonzalez de Peredo A (2012) Label-free quantification and shotgun analysis of complex proteomes by one-dimensional SDS-PAGE/NanoLC-MS: evaluation for

IPBS Institut de Pharmacologie et de Biologie Structurale
www.ipbs.fr
205 route de Narbonne
31077 Toulouse Cedex04
T. 33-5 61 17 59 18
F. 33-5 61 17 59 93
UMR 5089

the large scale analysis of inflammatory human endothelial cells. *Mol Cell Proteomics* **11:** 527-539

Geng F, Tansey WP (2012) Similar temporal and spatial recruitment of native 19S and 20S proteasome subunits to transcriptionally active chromatin. *Proc Natl Acad Sci U S A*

Grande A, Montanari M, Manfredini R, Tagliafico E, Zanocco-Marani T, Trevisan F, Ligabue G, Siena M, Ferrari S (2001) A functionally active RARalpha nuclear receptor is expressed in retinoic acid non responsive early myeloblastic cell lines. *Cell death and differentiation* **8:** 70-82

Guillaume B, Chapiro J, Stroobant V, Colau D, Van Holle B, Parvizi G, Bousquet-Dubouch MP, Theate I, Parmentier N, Van den Eynde BJ (2010) Two abundant proteasome subtypes that uniquely process some antigens presented by HLA class I molecules. *Proc Natl Acad Sci U S A* **107:** 18599-18604

Hamatani T, Carter MG, Sharov AA, Ko MS (2004) Dynamics of global gene expression changes during mouse preimplantation development. *Dev Cell* **6:** 117-131

Kaake RM, Kao A, Yu C, Huang L (2014) Characterizing the Dynamics of Proteasome Complexes by Proteomics Approaches. *Antioxid Redox Signal*

Kim MS, Pinto SM, Getnet D, Nirujogi RS, Manda SS, Chaerkady R, Madugundu AK, Kelkar DS, Isserlin R, Jain S, Thomas JK, Muthusamy B, Leal-Rojas P, Kumar P, Sahasrabuddhe NA, Balakrishnan L, Advani J, George B, Renuse S, Selvan LD et al (2014) A draft map of the human proteome. *Nature* **509:** 575-581

Kusmierczyk AR, Kunjappu MJ, Kim RY, Hochstrasser M (2011) A conserved 20S proteasome assembly factor requires a C-terminal HbYX motif for proteasomal precursor binding. *Nat Struct Mol Biol* **18:** 622-629

Malovannaya A, Lanz RB, Jung SY, Bulynko Y, Le NT, Chan DW, Ding C, Shi Y, Yucer N, Krenciute G, Kim BJ, Li C, Chen R, Li W, Wang Y, O'Malley BW, Qin J (2011) Analysis of the human endogenous coregulator complexome. *Cell* **145:** 787-799

Mouton-Barbosa E, Roux-Dalvai F, Bouyssie D, Berger F, Schmidt E, Righetti PG, Guerrier L, Boschetti E, Burlet-Schiltz O, Monsarrat B, Gonzalez de Peredo A (2010) In-depth exploration of cerebrospinal fluid by combining peptide ligand library treatment and label-free protein quantification. *Mol Cell Proteomics* **9:** 1006-1021

Murata S, Yashiroda H, Tanaka K (2009) Molecular mechanisms of proteasome assembly. *Nat Rev Mol Cell Biol* **10:** 104-115

Nagore LI, Nadeau RJ, Guo Q, Jadhav YL, Jarrett HW, Haskins WE (2013) Purification and characterization of transcription factors. *Mass spectrometry reviews* **32:** 386-398

iPBS Institut de Pharmacologie et de Biologie Structurale
www.ipbs.fr
205 route de Narbonne
31077 Toulouse Cedex04
T. 33-5 61 17 59 18
F. 33-5 61 17 59 93
UMR 5089

Nathan JA, Kim HT, Ting L, Gygi SP, Goldberg AL (2013) Why do cellular proteins linked to K63-polyubiquitin chains not associate with proteasomes? *The EMBO journal* **32:** 552-565

Picotti P, Aebersold R (2012) Selected reaction monitoring-based proteomics: workflows, potential, pitfalls and future directions. *Nature methods* **9:** 555-566

Picotti P, Bodenmiller B, Aebersold R (2013) Proteomics meets the scientific method. *Nature methods* **10:** 24-27

Silva JC, Gorenstein MV, Li GZ, Vissers JP, Geromanos SJ (2006) Absolute quantification of proteins by LCMSE: a virtue of parallel MS acquisition. *Mol Cell Proteomics* **5:** 144-156

Stadtmueller BM, Kish-Trier E, Ferrell K, Petersen CN, Robinson H, Myszka DG, Eckert DM, Formosa T, Hill CP (2012) Structure of a proteasome Pba1-Pba2 complex: implications for proteasome assembly, activation, and biological function. *The Journal of biological chemistry* **287:** 37371-37382

Tanahashi N, Murakami Y, Minami Y, Shimbara N, Hendil KB, Tanaka K (2000) Hybrid proteasomes. Induction by interferon-gamma and contribution to ATP-dependent proteolysis. *The Journal of biological chemistry* **275:** 14336-14345

Wang X, Huang L (2008) Identifying dynamic interactors of protein complexes by quantitative mass spectrometry. *Mol Cell Proteomics* **7:** 46-57

Yang XW, Wang P, Liu JQ, Zhang H, Xi WD, Jia XH, Wang KK (2014) Coordinated regulation of the immunoproteasome subunits by PML/RARalpha and PU.1 in acute promyelocytic leukemia. *Oncogene* **33:** 2700-2708

iPBS Institut de Pharmacologie et de Biologie Structurale
www.ipbs.fr
205 route de Narbonne
31077 Toulouse Cedex04
T. 33-5 61 17 59 18
F. 33-5 61 17 59 93
UMR 5089

2nd Editorial Decision 17 November 2014

Thank you again for submitting your work to Molecular Systems Biology. We have now heard back from the two referees who agreed to evaluate your manuscript. As you will see from the reports below, the referees are satisfied with the modifications made and they think that the study is now suitable for publication.

Reviewer #1:
The authors did a very good job and answered all the questions and concerns to my satisfaction.

Reviewer #2:
The authors have satisfactorily addressed my concerns. In fact, this is one of the most comprehensive responses to reviewers' concerns that I have seen.

© European Molecular Biology Organization
